# Towards a sequential microbiome therapy for HPV-associated cervicovaginal dysbiosis

**DOI:** 10.1038/s42003-026-11208-6

**Published:** 2026-10-29

**Authors:** Mariano A. Molina, Yichan Zhang, Renske D. M. Steenbergen, Willem J. G. Melchers, Juan Du

**Affiliations:** 1 Department of Pathology, Vrije Universiteit Amsterdam, Amsterdam UMC location VUmc, Amsterdam, The Netherlands; 2 Cancer Center Amsterdam, Imaging and Biomarkers, Amsterdam, The Netherlands; 3 Centre for Translational Microbiome Research (CTMR), Department of Microbiology, Tumor, and Cell Biology, Karolinska Institutet, Stockholm, Sweden; 4 Department of Medical Microbiology, Radboud University Medical Center, Nijmegen, The Netherlands; 5 Present Address: Department of Laboratory Medicine, Karolinska Institutet, ANA Futura, Stockholm, Sweden

**Keywords:** Human papilloma virus, Microbiome, Microbial ecology

## Abstract

Persistent high-risk human papillomavirus (hrHPV) infections are the principal cause of cervical cancer, and are increasingly associated with ecological instability of the cervicovaginal microbiome. Multiomics studies indicate that hrHPV persistence is linked to reduced *Lactobacillus* dominance, increased anaerobic taxa, and metabolic alterations that may compromise epithelial integrity and antiviral immunity. In this *Perspective* article, we introduce a sequential microbiome therapy (SMT) framework designed to restore cervicovaginal ecological balance and support viral clearance. SMT is a two-phase, strain-resolved strategy. The first phase targets dysbiotic communities using antimicrobials, biofilm-disrupting or microbiota-modulating approaches to create a window for ecological reset. The second phase introduces health-associated *Lactobacillus* species selected for their acidification capacity, competitive fitness, and ability to stabilize the mucosal environment. We outline the ecological principles underpinning SMT and discuss its development as a potentially scalable microbiome-directed strategy for restoring cervicovaginal health and managing hrHPV-associated disease within existing cervical cancer prevention frameworks.

## Introduction

Persistent high-risk human papillomavirus (hrHPV) infections in the cervical epithelium may lead to the development of cervical intraepithelial neoplasia (CIN), a precursor to cervical cancer (Box 1). HPV infection, CIN, and cervical cancer have been associated with an imbalanced cervicovaginal microbiome (CVM). In the current *Perspective*, we propose the concept of a sequential microbiome therapy (SMT) approach to promote cervicovaginal health and potentially assist the clearance of hrHPV infection and related disease. The SMT framework is primarily directed at early and intermediate stages of hrHPV-associated disease, where microbiome modulation may be most effective and clinically actionable. This strategy harnesses the dynamic^1^ and continuum nature^2,3^ of cervicovaginal microbial communities by employing *Lactobacillus* species as potential therapeutic agents against a detrimental CVM^4^. In this paper, we introduce the ecological and functional rationale behind SMT and compare it with existing approaches such as vaginal microbiome transplants (VMT), probiotics, and antibiotics^5^. We also urge the need for further research and clinical testing of this framework.

Box 1 hrHPV infection and cervical disease progression Persistent infection with hrHPV is the main cause of virtually all cervical cancer cases globally^106^. While most hrHPV infections are transient and cleared spontaneously by the immune system, persistent infections may lead to the development of CIN, which is graded according to the extent of epithelial dysplasia. CIN1 is considered a low-grade lesion with a high probability of spontaneous regression, whereas CIN2 and CIN3 are high-grade lesions associated with an increased risk of progression to cervical cancer^107^. Current management strategies are guided by cytology, HPV genotype, histopathology, and risk-based clinical guidelines, including those from the ASCCP and ESGO/ESTRO/ESP^108,109^. SMT is envisioned primarily as an adjunctive microbiome-directed strategy for women with persistent hrHPV infection, recurrent vaginal dysbiosis, or low-grade cervical abnormalities, and not as a replacement for established treatment of high-grade lesions.

### Current microbiome-directed interventions for cervicovaginal dysbiosis

Several microbiome-directed strategies have been explored to restore cervicovaginal health that could potentially be applied to reduce hrHPV persistence or disease progression. Current approaches include antibiotics, probiotics, live biotherapeutic products (LBPs), VMT, and emerging biofilm- or metabolite-targeted interventions^6,7^. Antibiotics such as metronidazole remain the standard treatment for bacterial vaginosis (BV) and can transiently reduce anaerobic dysbiotic taxa; however, recurrence rates remain high, and treatment frequently results in collateral depletion of beneficial *Lactobacillus* species^6,7^. Recently, combinations of antimicrobials with biofilm-disrupting or metabolite-based compounds, including oleic acid, have shown promise in destabilizing polymicrobial communities and improving ecological recovery (Table 1)^8,9^.

**Table 1:** Current microbiome-directed interventions for cervicovaginal dysbiosis

Strategy	Main principle	Advantages	Limitations	Relation to SMT
Antibiotics	Reduce anaerobic taxa	Rapid reduction of dysbiosis	High recurrence	Supports SMT phase 1 rationale
Probiotics/LBPs	Introduce beneficial *Lactobacillus*	Non-invasive	Poor engraftment	Supports SMT phase 2 rationale
VMT	Restore the whole microbial ecosystem	Broad ecological restoration	Donor dependence	SMT is donor-independent
Biofilm/metabolic modulation	Destabilize dysbiotic biofilms	May improve recolonization	Limited clinical evidence	Supports SMT phase 1
SMT	Sequential ecological disruption and restoration	Tailored, antibiotic-sparing ecological modulation	Requires validation	Proposed framework

Probiotics and LBPs aim to restore the cervicovaginal microbiome through the administration of selected strains, most commonly *Lactobacillus crispatus* or *Lactobacillus rhamnosus*^10–12^. Notably, studies evaluating LACTIN-V (*L. crispatus* CTV-05) demonstrated that vaginal engraftment of *L. crispatus* can reduce BV recurrence and support longer-term microbiome stabilization, providing important evidence for ecological restoration strategies^13–16^. However, colonization success remains variable across studies and individuals, and many probiotic formulations rely on strains that are not specifically adapted to the vaginal niche. VMT has also emerged as a potential strategy for restoring complex microbial communities in women with recurrent dysbiosis^17,18^. While promising, VMT remains donor-dependent and raises challenges related to donor screening, standardization, long-term safety, and regulatory implementation. Importantly, recent randomized controlled data evaluating VMT without antibiotic pretreatment showed limited efficacy overall, but antiseptic pretreatment before VMT improved microbiome conversion and donor engraftment, supporting the importance of ecological disruption before stable microbial restoration^17^. Collectively, these findings suggest that durable microbiome modulation may require both disruption of resilient dysbiotic communities and stable recolonization with functionally adapted vaginal strains. SMT builds on this ecological principle by proposing a sequential strategy that first destabilizes detrimental microbial states and subsequently promotes long-term cervicovaginal stability through targeted *Lactobacillus* engraftment (Table 1).

### Cervicovaginal microbiome dynamics and correlation with hrHPV-related disease

The CVM plays a pivotal role in maintaining cervicovaginal health, and a fundamental aspect of SMT is based on the understanding of the composition and dynamics of cervicovaginal microbial communities^1,19^. The CVM can be clustered into community state types (CSTs) according to its composition, with *Lactobacillus crispatus*, *Lactobacillus gasseri*, *Lactobacillus iners*, and *Lactobacillus jensenii* dominating the CSTs I, II, III, and V, respectively, and maintaining an acidic pH (3.5-4.5) in the microenvironment^2,19–22^. Although most *Lactobacillus*-rich communities are associated with healthy conditions, *L. iners*-dominated microbiomes (CST III) have been the exception and exhibit a higher risk for dysbiosis and infections^23^. Alternatively, highly diverse microbial (polymicrobial) communities (CST IV) are characterized by higher vaginal pH ( > 4.5) and *Lactobacillus* depletion^5,24,25^. Additionally, more detailed CVM state types have been proposed recently, including VALENCIA^19^ and metagenomic community state types (mgCSTs)^26^, the latter emphasizing the different roles of bacterial genes in the biology and ecology of the CVM^19,26,27^.

Our research, together with others, demonstrated the dynamics of the CVM, which can transition from one CST to another CST in 28–30 days^1,28–30^. We also recently proposed the classification framework Vaginal Community Dynamics (VCD), which allows a deeper understanding and estimation of the CVM dynamics, and the initiation and maintenance of dysbiosis^1,31^. These dynamics are influenced by several intrinsic and external factors, including age, ethnicity, hygiene, menstrual cycle, and sexual activities^1,32–37^. Importantly, eubiotic cervicovaginal communities may differ substantially across populations and ethnic groups^38^. Several studies have shown that some women, particularly those from African and Latin American populations, may exhibit more diverse microbial communities without apparent symptoms or disease^36,39^. These findings suggest that cervicovaginal eubiosis likely exists along a broader ecological continuum. Consequently, similar to other strategies, SMT strategies may ultimately require adaptation to population-specific microbial baselines, host genetics, environmental exposures, and local hrHPV genotype prevalence to avoid oversimplified therapeutic approaches^39^.

Behavioral and hygiene-associated practices may also contribute to microbiome instability and should be considered in future SMT-oriented clinical management strategies^40,41^. Because estrogen-associated glycogen deposition may promote *Lactobacillus* dominance, timing the recolonization phase of SMT to specific phases of the menstrual cycle may improve engraftment efficiency and long-term microbiome stability^1,42^. Recent evidence also suggests that treatment of male sexual partners may reduce BV recurrence^43^, highlighting the potential contribution of sexual transmission and shared microbial reservoirs to long-term cervicovaginal microbiome stability^43,44^. These findings further support the importance of ecological approaches aimed at achieving durable microbial restoration.

Although numerous longitudinal studies have demonstrated that cervicovaginal dysbiosis precedes and predicts hrHPV persistence and cervical disease progression^45–47^, definitive causal relationships remain to be established. It remains unclear whether dysbiosis directly promotes viral persistence, arises as a consequence of HPV infection, or reflects bidirectional interactions between the virus, host, and microbial ecosystem.

Women with hrHPV clearance and disease regression are often characterized by stable CSTs I, II, or V, and women with persistent hrHPV infections and high-grade CIN are often associated with CST IV and transitions from *Lactobacillus*-rich communities towards a *Lactobacillus*-depleted community^28,46–50^. *L. iners-*dominated CVM has also been associated with hrHPV acquisition and persistence^34,51,52^. Notably, hrHPV induces several microenvironmental changes that reshape microbial ecology. hrHPV downregulates key epithelial innate peptides (e.g., S100A7 and elafin) that *Lactobacillus* species rely on as nutrient sources^47^, reducing their competitiveness and facilitating transitions toward polymicrobial states. In parallel, lactic acid isomers produced by different *Lactobacillus* species differentially regulate epithelial signaling pathways involved in stem cell self-renewal and early transformation, including YAP1- and PI3K-AKT-associated responses^53^. Additional microbial metabolites, including short-chain fatty acids (SCFAs), further modulate local immune responses and epithelial homeostasis within the cervicovaginal niche^8,54,55^. Dysbiotic communities may also contribute to hrHPV persistence by disrupting epithelial barrier integrity and promoting a pro-inflammatory milieu^56–58^. Studies have shown that *Sneathia vaginalis* can induce epithelial damage and stress responses that may increase susceptibility to hrHPV persistence, whereas loss of *Lactobacillus crispatus*-derived protective vesicles involved in epithelial repair and mucosal homeostasis may further contribute to a microenvironment permissive to viral persistence^32,59,60^. Furthermore, emerging evidence, including data from our group, suggests that different hrHPV genotypes, such as 16 and 18, are associated with distinct microbial functional and metabolic profiles^61,62^. These genotype-linked differences may reflect variation in viral oncogenic potential and disease severity but collectively indicate that HPV genotype could contribute to shaping the ecological and metabolic landscape of the cervicovaginal niche^61–64^. These mechanisms support the concept that hrHPV-microbiome interactions are functional and bidirectional rather than purely compositional^65^.

### Strain-level specificity

**Fig. 1:**
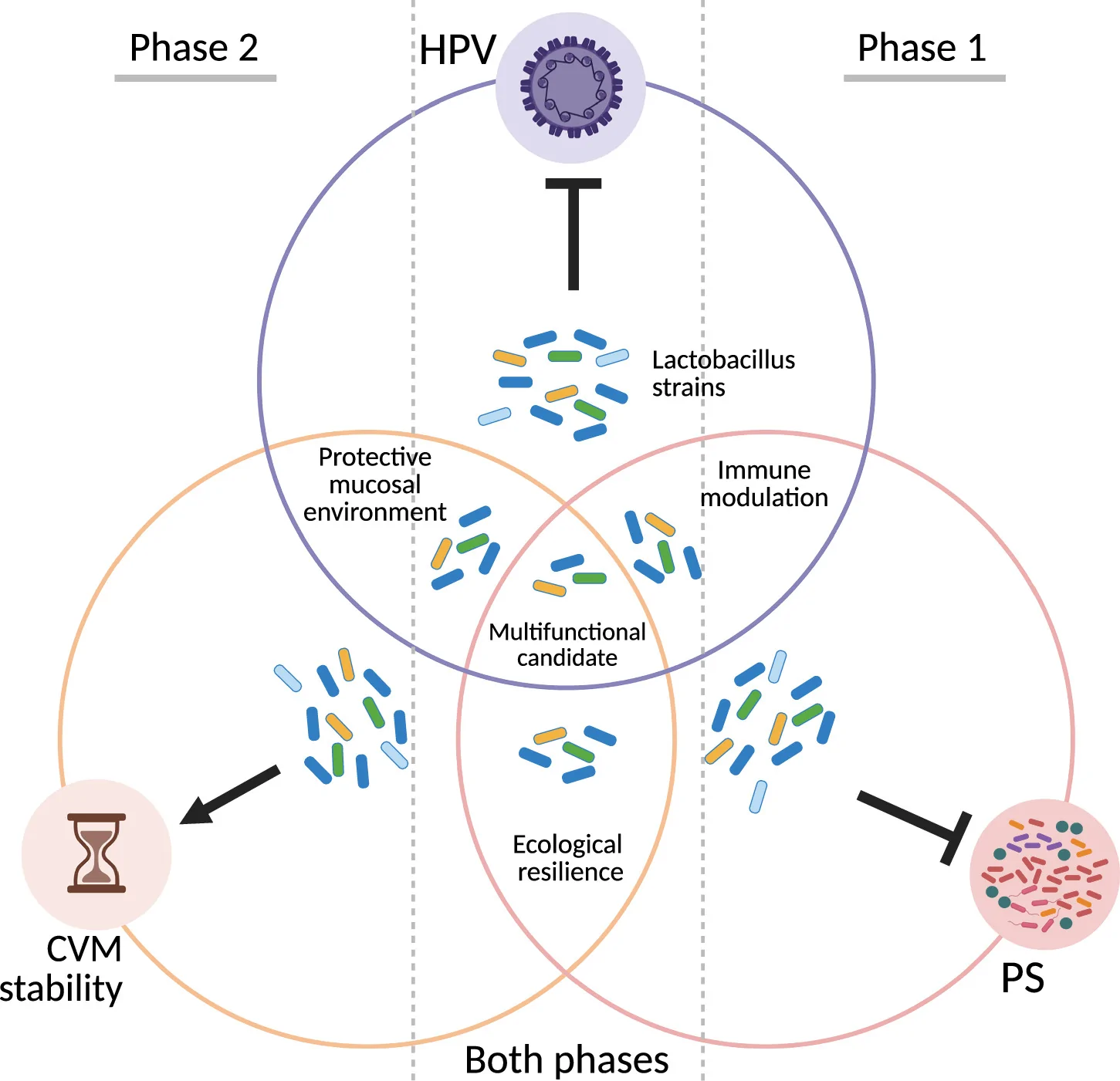
Schematic representation of functional *Lactobacillus* properties relevant to SMT. *Lactobacillus* strains’ (from e.g., *L. crispatus, L. gasseri*, or *L. jensenii*) functional activities may differ in promoting stability in the cervicovaginal microbiome (CVM), inhibiting a polymicrobial state (PS), and suppressing human papillomavirus (HPV). Strains with strong competitive or inhibitory activity may be particularly useful during ecological disruption in Phase 1, whereas strains with high colonization fitness and capacity to promote long-term microbiome stability may be prioritized during Phase 2. Strains with all three properties represent multifunctional candidates that could contribute to one or both phases of a sequential microbiome therapy (SMT). The left and right regions separated by gray dashed lines indicate functions that may be particularly relevant to Phase 2 and Phase 1, respectively, whereas overlapping regions represent properties that may contribute to both phases.

With the growing evidence of sequencing data on the CVM at the bacterial species- or strain-levels, including strain-level comparisons on phenotypes and genotypes, different strains from the same *Lactobacillus* species have been shown to exhibit strain-specific features^26,66^. This genomic diversity and unique characteristics within *Lactobacillus* species are believed to play a key functional role in the CVM. For example, distinctive *L. crispatus* strains exhibit phenotypic and genomic variations as well as antimicrobial and inflammatory properties, which allow them to adjust to different microenvironments, thereby influencing their colonization efficiency and the treatment outcomes against BV^1,4,5,26,28,67–71^. Notably, strain-level specificity builds on the broader principle that *Lactobacillus* species are not functionally interchangeable across body sites. Vaginal *Lactobacillus* species differ markedly from gut-associated strains in their metabolic repertoire, ecological fitness, and host-microbe interactions, which partly explains the limited success of early probiotic trials relying on non-vaginal isolates^4,72^. Even among vaginal species, *L. crispatus*-dominated communities show greater longitudinal stability than *L. gasseri* or *L. iners*, and strain-level genomic variation in pathways such as glycogen utilization, glycosylation, and antimicrobial activity can critically influence colonization success and therapeutic efficacy^26,28,67,71^. Thus, due to the functional role of strain-level diversity, we postulate that microbiome therapies for cervicovaginal diseases, especially those aimed at counteracting hrHPV infection and persistence, should consider the ecological dynamics of the CVM at both species- and strain-level functions (Fig. 1)^12,28,73–76^. Importantly, therapies should aim to restore a stable CST I, II, or V while inhibiting the (shift towards) risky CST III and the detrimental CST IV. SMT takes this into account by considering strain-level interactions and the unique microbial community structure. This tailored approach enables the selection of not just the strains that are the best hrHPV inhibitors but also strains that can help maintain a healthy microbiome, preventing dysbiosis and supporting long-term cervicovaginal health (Fig. 1).

### The approach of a sequential microbiome therapy

The SMT framework proposes a two-phase treatment strategy tailored to the specific detrimental or risk microbial community of each individual, with the first phase involving destabilizing the existing microbial community. SMT is envisioned primarily as a vaginally administered intervention, potentially delivered through gels, capsules, sprays, or other locally applied formulations. Current antibiotic treatments against BV, such as metronidazole, would achieve this at the cost of inhibiting beneficial microbes^4,77,78^. However, this could also be achieved by using biofilm-disrupting agents, lactic acids, or oleic acid (Fig. 2.1a)^8,34^, which are known to enhance the cervicovaginal epithelial barrier integrity, ameliorate cytotoxic inflammatory responses, and inhibit dysbiosis-related bacteria. Experimental and clinical studies have shown that biofilm-disrupting agents, including surfactants, extracellular matrix-disrupting enzymes, amphipathic molecules, and polymer-based agents, can destabilize the biofilm architecture, improve antimicrobial penetration, and selectively reduce pathogenic taxa while preserving or facilitating recolonization by *Lactobacillus*^9,79,80^. Although antibiotics may be incorporated into Phase 1, SMT prioritizes targeted biofilm-disrupting, metabolic, or microbiota-based strategies where feasible, with the aim of minimizing collateral disruption of beneficial *Lactobacillus* populations and reducing reliance on repeated antibiotic treatment.

**Fig. 2:**
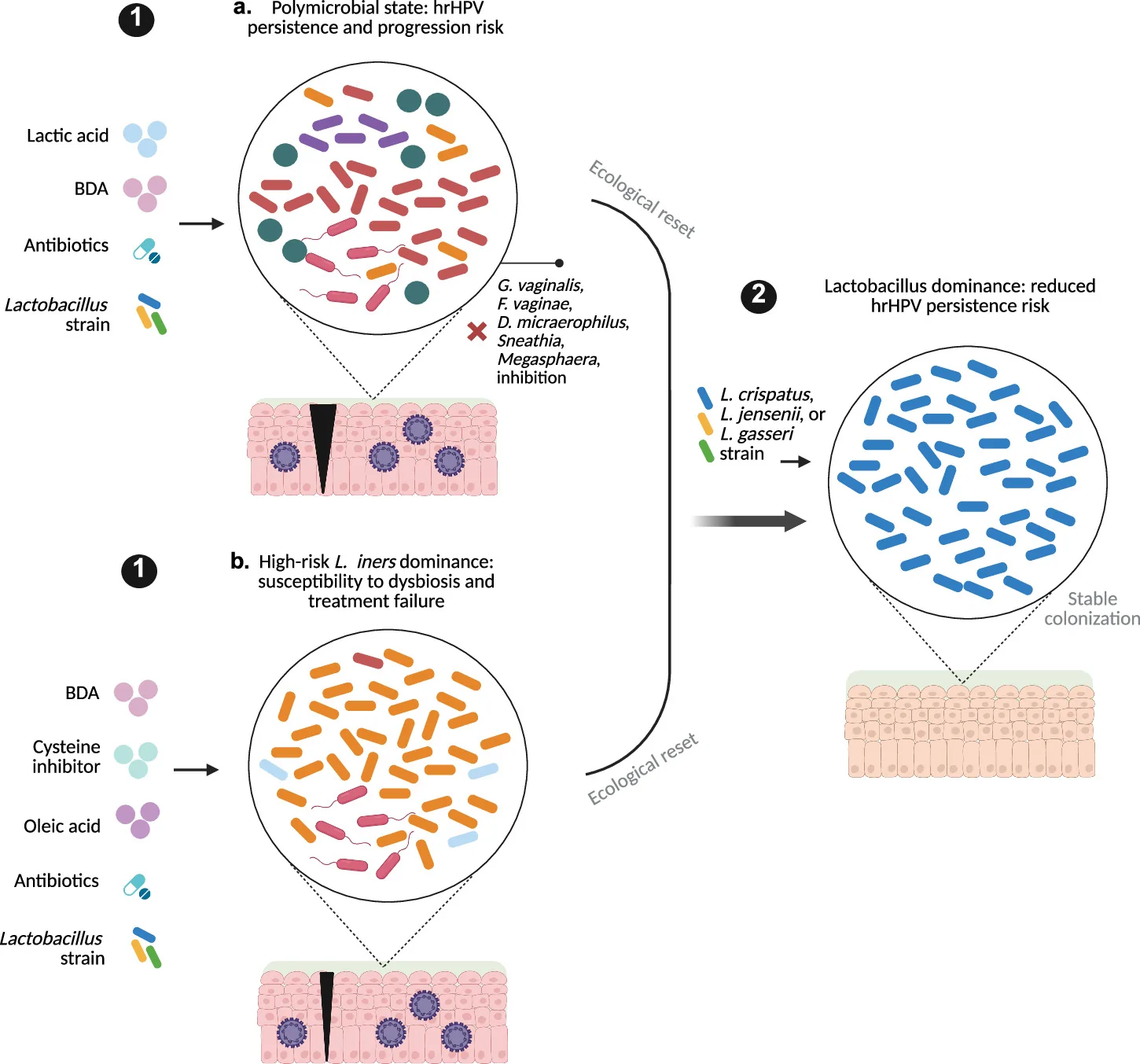
Sequential microbiome therapy (SMT) for treating hrHPV-related cervicovaginal dysbiosis. SMT employs a targeted two-phase treatment. The first phase (**1a** or **1b** for a baseline polymicrobial or *L. iners*-dominated community, respectively) uses compounds or bacterial strains with inhibitory activity to disrupt detrimental microbial communities. This shifts the microbiome from a detrimental/risk community to a microenvironment that is beneficial for *Lactobacilli* by inhibiting *Gardnerella vaginalis, Fannyhessea vaginae, Dialister micraerophilus, Prevotella, and Megasphaera*, which are associated with stable detrimental communities. The second phase (**2**) aims to establish a stable, healthy *Lactobacillus*-dominant environment using strains of *L. crispatus*, *L. gasseri*, *or L. jensenii*. This approach addresses the dynamic nature of the CVM and is tailored to the individual’s specific microbial community. BDA, biofilm-disrupting agents.

In this step, we emphasize that inhibiting the CVM bacteria, such as *Gardnerella vaginalis*, *Fannyhessea vaginae*, *Dialister micraerophilus*, *Prevotella*, and *Megasphaera*, which can harm the vaginal epithelium and maintain a stable polymicrobial state, is essential^28,81^. hrHPV may also attenuate epithelial damage caused by specific dysbiosis-associated bacteria, allowing these taxa to persist and maintain a pro-inflammatory microenvironment^60^. Disrupting these virus-bacterium feedback loops is therefore a critical goal of the first phase of SMT. *Lactobacillus* species have also shown promising effects on inhibiting these bacterial species in vitro^82,83^. We believe certain *Lactobacillus s*pecies and strains may have better inhibition ability towards dysbiosis-related bacteria than others (Fig. 2). Strains that efficiently lower the vaginal pH and inhibit dysbiosis-related species may therefore represent promising candidates for the first phase of SMT. For women with a high-risk *L. iners*-dominated community (CST III), the first phase involves inhibiting *L. iners* (Fig. 2.1b). This could be achieved by using biofilm-disrupting agents, cysteine inhibitors, or oleic acid^8,23^. *L. iners* growth is highly dependent on the presence of L-cysteine and the absence of oleic acid in the cervicovaginal microenvironment. The bacterium uses L-cysteine as an amino acid source and lacks oleic acid-induced genes, making it less competitive against the growth of other *Lactobacilli*^8,23^. The subsequent disruption of polymicrobial states or *L. iners-*dominance aims to create an environment that is beneficial for the growth of other *Lactobacillus* species. Based on their distinct ecological and functional properties, different *Lactobacillus* species may also be preferentially suited for different phases of SMT. For example, *L. gasseri* and *L. jensenii* strains may facilitate early ecological transition through rapid acidification, competitive exclusion of dysbiosis-associated taxa, and immune modulation^84–86^, whereas *L. crispatus* may be particularly advantageous during the restoration phase because of its strong epithelial adherence, long-term stability, and association with durable *Lactobacillus*-dominant communities^13–15^. Nevertheless, synergistic or complementary combinations of strains may also be effective and require further investigation. Importantly, the distinction between Phase 1 and Phase 2 is functional and not strictly based on intervention type; strains used in Phase 1 would be selected primarily for their ability to disrupt dysbiosis-associated communities, whereas Phase 2 would prioritize durable engraftment and long-term ecological stability. Although individual strains may possess properties relevant to both phases, their selection criteria and intended function would differ between Phase 1 and Phase 2.

The second phase of SMT involves transitioning to a stable *Lactobacillus*-rich environment and avoiding the undesirable *L. iners*-dominated or low-*Lactobacilli* CVM. This is accomplished by administering a second treatment containing *L. crispatus*, *L. gasseri*, or *L. jensenii* strains that exert strong adherence to cervicovaginal epithelial cells, support epithelial barrier integrity, and antiviral host responses (Fig. 2.2)^83,85,87^. The goal of SMT is therefore to promote microbial transitions towards stable health-associated communities (e.g., CSTs I, II, or V) containing effective and long-lasting *Lactobacillus* strains, with the potential for activation of the immune system, thereby assisting hrHPV clearance^28^. In particular, *L. gasseri* strains have been associated with the lowest risk of hrHPV infection as well as with reduced viral persistence and modulation of local immune responses, suggesting it could be used against hrHPV^37,85,88,89^.

SMT is especially important for hrHPV-associated dysbiosis because it allows for a nuanced approach to microbiome modulation that helps to address the complexities of hrHPV-related disease. While certain *Lactobacillus* strains may exert stronger abilities to inhibit hrHPV, focusing solely on these strains might overlook the broader ecological dynamics of the CVM^1^. For example, a strain that effectively produces lactic acid and lowers pH may not necessarily be the best choice for inhibiting hrHPV if it does not also outcompete other harmful bacteria or support overall microbiome stability (Fig. 1). Thus, SMT may support a cervicovaginal microenvironment associated with viral clearance by promoting a balanced microbiome that can sustainably manage hrHPV infection and reduce the risk of cervical cancer development. By sequentially disrupting detrimental communities and reintroducing functionally selected *Lactobacillus* strains, SMT may help modulate genotype-specific ecological pressures and promote longer-term microbial stability supportive of hrHPV clearance. Because the optimal combinations of microbial strains and ecological modulators remain unknown, SMT should currently be viewed as a conceptual framework and not as a fixed therapeutic formulation.

### Advantages and limitations of a sequential microbiome therapy

SMT offers several advantages over other CVM therapies. SMT is a targeted approach, focusing on key bacterial species and strains associated with microbiome stability and hrHPV clearance^4^. The strategy is also donor-independent and could be examined for potential transferability of antibiotic resistance genes, virulence-associated traits, and infectious risks before use (Table 1). Furthermore, this strategy may achieve lasting changes and prevent dysbiosis recurrence^4,7,21,90,91^, potentially decreasing the need for repeated antibiotic treatment. Although antibiotics remain effective for managing BV, they may disrupt beneficial *Lactobacillus* populations and contribute to selective pressure for antimicrobial resistance^6,7^. SMT offers a complementary, antibiotic-sparing ecological approach that prioritizes targeted microbiome modulation and restoration, while allowing antibiotics to be incorporated when clinically or ecologically appropriate.

Notably, a recent combination of the antibiotic metronidazole with the compound oleic acid was found to be successful in inhibiting the vaginal polymicrobial state, which supports the notion of an SMT strategy and suggests that a combined administration of antibiotics with other inhibitors might be useful against hrHPV-associated dysbiosis^8^. Another advantage of SMT is the ease of cultivation and scalability in production. The components can be easily distributed worldwide, including countries with limited medical care resources, such as low- and middle-income countries^66^. Recent preclinical and translational data indicate that vaginal *L. crispatus* strains isolated from populations in South Africa and the U.S. show comparable colonization capacity and functional activity across geographic settings, suggesting that effective vaginal colonization is not country- or population-specific^92^. The selected strains could also be employed to develop low-cost cream-, gel-, or spray-based formulations that can be applied sequentially by women themselves at home^12,73,74,93–95^.

In terms of the sequential strategy, the selected strains will have strong antiviral and immune-modulating properties, and adding them sequentially may maximize the chances of utilizing the best features from each strain. This approach can help them outcompete dysplasia-related bacteria without the selective pressure associated with antibiotic treatment and ensure better adhesion to the epithelium and protection of the mucus layer and epithelial cells^96^. Adding the strains sequentially may help harness the natural ecological dynamics of the CVM and create the best microenvironment for long-lasting effects. Taken together, by leveraging the dynamic nature of cervicovaginal microbial communities, SMT represents a promising and targeted approach to support cervicovaginal health in the context of hrHPV-associated disease, warranting further investigation through comprehensive research and clinical studies.

### Outlook

While an SMT approach holds promise for treating hrHPV-related disease and potentially other adverse cervicovaginal conditions such as BV, preterm birth, and fungal infections, its feasibility and efficacy must be tested through in vitro and in vivo experiments. Comparative studies with other CVM therapies, including VMT and probiotics, should be conducted to assess their relative benefits and drawbacks^5,6,97^. Furthermore, research efforts should explore the potential of SMT in assisting clearance of persistent HPV infections in clinical testing and its long-term effects on women’s health. SMT is envisioned for women with persistent hrHPV infection, hrHPV-positive women with mild cytological abnormalities who do not yet require treatment, and women with recurrent cervicovaginal dysbiosis co-occurring with hrHPV^98,99^. Rather than replacing standard-of-care management for CIN, SMT is intended as an adjunctive, microbiome-directed strategy during the early phase between hrHPV detection and potential lesion development.

Residual biofilm architecture and ecological resilience also limit engraftment of introduced *Lactobacillus* strains following the first phase of SMT. Thus, combining SMT with metabolic priming approaches, including glycogen supplementation or human milk oligosaccharides, may enhance colonization success and ecological stability^100–102^. Longitudinal microbiome sampling may ultimately be necessary to optimize SMT timing, monitor ecological transitions, and evaluate long-term microbiome stability. Of note, in Europe and other regulatory jurisdictions, vaginal *Lactobacillus*-based formulations intended for disease treatment are classified as LBPs or biological medicinal products, which may require GMP-grade manufacturing and extensive clinical validation before regulatory approval^103,104^. These requirements drastically increase development costs and represent an important translational challenge for SMT implementation. The administration of live *Lactobacillus* strains also requires strain-level safety assessment, including potential horizontal gene transfer and antimicrobial resistance genes. However, rare cases of invasive infection associated with probiotic *Lactobacillus* administration have been reported, particularly in vulnerable populations, supporting appropriate safety monitoring during clinical development^105^. Similar to other strategies, SMT ultimately requires adaptive or personalized implementation strategies that account for temporal microbiome fluctuations associated with aging, hormonal transitions, sexual behavior, and persistent hrHPV infection.

SMT represents a conceptual ecological framework for microbiome-directed modulation of hrHPV-associated cervicovaginal dysbiosis. The integration of ecological disruption, strain-level specificity, and long-term microbial stabilization may offer a complementary strategy to support cervicovaginal health in women with persistent hrHPV infection. Future longitudinal and interventional studies will be essential to determine the safety, feasibility, and clinical utility of this approach.

## Supplementary information

Transparent Peer Review file

## Data Availability

No datasets were generated or analyzed for this *Perspective*.

## References

[1] Hugerth, L. W. et al. Defining vaginal community dynamics: daily microbiome transitions, the role of menstruation, bacteriophages, and bacterial genes. *Microbiome* **12**, 153 10.1186/s40168-024-01870-5 (2024). PMC11331738 39160615

[2] Lebeer, S. et al. A citizen-science-enabled catalogue of the vaginal microbiome and associated factors. *Nat. Microbiol.* **8**, 2183–2195 10.1038/s41564-023-01500-0 (2023). PMC10627828 37884815

[3] Chen, C. et al. The microbiota continuum along the female reproductive tract and its relation to uterine-related diseases. *Nat. Commun.* **8**, 875 10.1038/s41467-017-00901-0 (2017). PMC5645390 29042534

[4] Wu, S., Hugerth, L. W., Schuppe-Koistinen, I. & Du, J. The right bug in the right place: opportunities for bacterial vaginosis treatment. *npj Biofilms Microbiomes* **8**, 34 10.1038/s41522-022-00295-y (2022). PMC9061781 35501321

[5] Mitra, A. et al. Genital tract microbiota composition profiles and use of prebiotics and probiotics in gynaecological cancer prevention: review of the current evidence, the European Society of Gynaecological Oncology prevention committee statement. *Lancet Microbe* **5**, e291–e300 10.1016/S2666-5247(23)00257-4 (2024). 38141634

[6] Lev-Sagie, A. et al. Vaginal microbiome transplantation in women with intractable bacterial vaginosis. *Nat. Med.* **25**, 1500–1504 10.1038/s41591-019-0600-6 (2019). 31591599

[7] Gustin, A. T. et al. Recurrent bacterial vaginosis following metronidazole treatment is associated with microbiota richness at diagnosis. *Am. J. Obstet. Gynecol.* **226**, 225.e221–225.e215 10.1016/j.ajog.2021.09.018 (2022). PMC8887553 34560047

[8] Zhu, M. et al. Vaginal Lactobacillus fatty acid response mechanisms reveal a metabolite-targeted strategy for bacterial vaginosis treatment. *Cell* **187**, 5413–5430.e5429 10.1016/j.cell.2024.07.029 (2024). PMC11429459 39163861

[9] Gao, M., Manos, J., Whiteley, G. & Zablotska-Manos, I. Antibiofilm agents for the treatment and prevention of bacterial vaginosis: a systematic narrative review. *J. Infect. Dis.* **230**, e508–e517 10.1093/infdis/jiae134 (2024). PMC11420799 38680027

[10] Palma, E. et al. Long-term Lactobacillus rhamnosus BMX 54 application to restore a balanced vaginal ecosystem: a promising solution against HPV-infection. *BMC Infect. Dis.* **18**, 13 10.1186/s12879-017-2938-z (2018). PMC5756375 29304768

[11] Bertuccini, L., Russo, R., Iosi, F. & Superti, F. Effects of Lactobacillus rhamnosus and Lactobacillus acidophilus on bacterial vaginal pathogens. *Int. J. Immunopathol. Pharmacol.* **30**, 163–167 10.1177/0394632017697987 (2017). PMC5806794 28580872

[12] Oerlemans, E. F. M. et al. Impact of a lactobacilli-containing gel on vulvovaginal candidosis and the vaginal microbiome. *Sci. Rep.* **10**, 7976 10.1038/s41598-020-64705-x (2020). PMC7224289 32409699

[13] Cohen, C. R. et al. Randomized trial of lactin-V to prevent recurrence of bacterial vaginosis. *N. Engl. J. Med.* **382**, 1906–1915 10.1056/NEJMoa1915254 (2020). PMC7362958 32402161

[14] Armstrong, E. et al. Sustained effect of LACTIN-V (Lactobacillus crispatus CTV-05) on genital immunology following standard bacterial vaginosis treatment: results from a randomised, placebo-controlled trial. *Lancet Microbe* **3**, e435–e442 10.1016/S2666-5247(22)00043-X (2022). PMC9188188 35659905

[15] Bloom, S. M. et al. Vaginal microbiota impacts of a Lactobacillus crispatus live biotherapeutic and predictors of colonization in randomized controlled trial. *Cell Host Microbe* **34**, 734–750.e710 10.1016/j.chom.2026.03.003 (2026). PMC13525762 41903526

[16] Hemmerling, A., Wierzbicki, M. R., Armstrong, E. & Cohen, C. R. Response to antibiotic treatment of bacterial vaginosis predicts the effectiveness of LACTIN-V (Lactobacillus crispatus CTV-05) in the prevention of recurrent disease. *Sexually Transmit. Dis.* **51**, 437–440 10.1097/olq.0000000000001962 (2024). PMC11090451 38733973

[17] Wrønding, T. et al. Vaginal microbiota transplantation for treatment of vaginal dysbiosis without the use of antibiotics: a double-blind, randomised controlled trial in women with vaginal dysbiosis. *Lancet Microbe* **7** 10.1016/j.lanmic.2025.101294(2026). 41905375

[18] Wrønding, T. et al. Antibiotic-free vaginal microbiota transplant with donor engraftment, dysbiosis resolution and live birth after recurrent pregnancy loss: a proof of concept case study. *eClinicalMedicine* **61**, 102070 10.1016/j.eclinm.2023.102070 (2023). PMC10388571 37528843

[19] France, M. T. et al. VALENCIA: a nearest centroid classification method for vaginal microbial communities based on composition. *Microbiome* **8**, 166 10.1186/s40168-020-00934-6 (2020). PMC7684964 33228810

[20] Molina, M. A., Andralojc, K. M., Huynen, M. A., Leenders, W. P. J. & Melchers, W. J. G. In-depth insights into cervicovaginal microbial communities and hrHPV infections using high-resolution microbiome profiling. *npj Biofilms Microbiomes* **8**, 75 10.1038/s41522-022-00336-6 (2022). PMC9519886 36171433

[21] France, M., Alizadeh, M., Brown, S., Ma, B. & Ravel, J. Towards a deeper understanding of the vaginal microbiota. *Nat. Microbiol.* **7**, 367–378 10.1038/s41564-022-01083-2 (2022). PMC8910585 35246662

[22] Ravel, J. et al. Vaginal microbiome of reproductive-age women. *Proc. Natl. Acad. Sci. USA* **108**, 4680–4687 10.1073/pnas.1002611107 (2011). PMC3063603 20534435

[23] Bloom, S. M. et al. Cysteine dependence of Lactobacillus iners is a potential therapeutic target for vaginal microbiota modulation. *Nat. Microbiol.* **7**, 434–450 10.1038/s41564-022-01070-7 (2022). PMC10473153 35241796

[24] Norenhag, J. et al. The vaginal microbiota, human papillomavirus and cervical dysplasia: a systematic review and network meta-analysis. *BJOG: Int. J. Obstet. Gynaecol.* **127**, 171–180 10.1111/1471-0528.15854 (2020). 31237400

[25] Usyk, M. et al. Cervicovaginal microbiome and natural history of Chlamydia trachomatis in adolescents and young women. *Cell*10.1016/j.cell.2024.12.011 (2025). PMC12035847 39818212

[26] Holm, J. B. et al. Integrating compositional and functional content to describe vaginal microbiomes in health and disease. *Microbiome* **11**, 259 10.1186/s40168-023-01692-x (2023). PMC10688475 38031142

[27] Moosa, Y., Kwon, D., de Oliveira, T. & Wong, E. B. Determinants of vaginal microbiota composition. *Front. Cell. Infect. Microbiol.* **10**, 467 (2020). 10.3389/fcimb.2020.00467 PMC7492712 32984081

[28] Molina, M. A., Melchers, W. J. G., Andralojc, K. M., Leenders, W. P. J. & Huynen, M. A. Longitudinal analysis on the ecological dynamics of the cervicovaginal microbiome in hrHPV infection. *Comput. Struct. Biotechnol. J.* **21**, 4424–4431 10.1016/j.csbj.2023.09.011 (2023). PMC10507478 37731597

[29] Mejia, M. E. et al. Vaginal microbial dynamics and pathogen colonization in a humanized microbiota mouse model. *npj Biofilms Microbiomes* **9**, 87 10.1038/s41522-023-00454-9 (2023). PMC10661851 37985659

[30] Munoz, A. et al. Modeling the temporal dynamics of cervicovaginal microbiota identifies targets that may promote reproductive health. *Microbiome* **9**, 163 10.1186/s40168-021-01096-9 (2021). PMC8314590 34311774

[31] Malave-Ortiz, S., Calvert, M. E., Hood-Pishchany, I. & Shaw, N. D. The vaginal microbiome and reproductive health in adolescents and adults. *J. Pediatric Adolescent Gynecol.* 10.1016/j.jpag.2024.12.008(2024). PMC11875951 39667660

[32] Wei, X. et al. Vaginal microbiomes show ethnic evolutionary dynamics and positive selection of Lactobacillus adhesins driven by a long-term niche-specific process. *Cell Rep.* **43**, 114078 10.1016/j.celrep.2024.114078 (2024). 38598334

[33] Lee, C. Y., Dillard, L. R., Papin, J. A. & Arnold, K. B. New perspectives into the vaginal microbiome with systems biology. *Trends Microbiol.* **31**, 356–368 10.1016/j.tim.2022.09.011 (2023). 36272885

[34] Molina, M. A. et al. Assessing the cervicovaginal microbiota in the context of hrHPV infections: temporal dynamics and therapeutic strategies. *mBio* **13**, e01619–22 10.1128/mbio.01619-22 (2022). PMC9600249 35980030

[35] Morsli, M. et al. The association between lifestyle factors and the composition of the vaginal microbiota: a review. *Eur. J. Clin. Microbiol. Infect. Dis.* **43**, 1869–1881 10.1007/s10096-024-04915-7 (2024). PMC11405494 39096320

[36] Mancilla, V., Jimenez, N. R., Bishop, N. S., Flores, M. & Herbst-Kralovetz, M. M. The vaginal microbiota, human papillomavirus infection, and cervical carcinogenesis: a systematic review in the latina population. *J. Epidemiol. Glob. Health* **14**, 480–497 10.1007/s44197-024-00201-z (2024). PMC11176136 38407720

[37] Łaniewski, P., Ilhan, Z. E. & Herbst-Kralovetz, M. M. The microbiome and gynaecological cancer development, prevention and therapy. *Nat. Rev. Urol.* **17**, 232–250 10.1038/s41585-020-0286-z (2020). PMC9977514 32071434

[38] Sun, S. et al. Race, the vaginal microbiome, and spontaneous preterm birth. *mSystems* **7**, e00017–22 10.1128/msystems.00017-22 (2022). PMC9238383 35582911

[39] Condori-Catachura, S. et al. Diversity in women and their vaginal microbiota. *Trends Microbiol.* **33**, 1163–1172 10.1016/j.tim.2024.12.012 (2025). PMC12628047 39919958

[40] Brown, S. E. et al. Douching cessation and molecular bacterial vaginosis: a reanalysis of archived specimens. *Sexually Transmitted Infect.* **99**, 156 10.1136/sextrans-2022-055459 (2023). PMC9708945 35636931

[41] Hesham, H., Mitchell, A. J., Bergerat, A., Hung, K. & Mitchell, C. M. Impact of vaginal douching products on vaginal Lactobacillus, Escherichia coli and epithelial immune responses. *Sci. Rep.* **11**, 23069 10.1038/s41598-021-02426-5 (2021). PMC8629978 34845288

[42] Vinerbi, E. et al. Lactobacillus iners dominates the vaginal microbiota of healthy Italian women of reproductive age. *mSystems* **10**, e00983–25 10.1128/msystems.00983-25 (2025). PMC12710342 41288406

[43] Vodstrcil, L. A. et al. Male-partner treatment to prevent recurrence of bacterial vaginosis. *N. Engl. J. Med.* **392**, 947–957 10.1056/NEJMoa2405404 (2025). 40043236

[44] Yazdy, G. M. et al. Male partner treatment and the potential sexual transmission of bacterial vaginosis: considerations for patient counseling and clinical application in the United States. *Am. J. Obstet. Gynecol.* **234**, 606–612 10.1016/j.ajog.2025.11.001 (2026). 41213492

[45] Molina, M. A. From dysbiosis to mechanisms: Why cervicovaginal microbiome-HPV studies must catch up with biology. *PLoS Pathog.* **21**, e1013830 10.1371/journal.ppat.1013830 (2026). PMC12752938 41468410

[46] Molina, M. A., Leenders, W. P. J., Huynen, M. A., Melchers, W. J. G. & Andralojc, K. M. Temporal composition of the cervicovaginal microbiome associates with hrHPV infection outcomes in a longitudinal study. *BMC Infect. Dis.* **24**, 552 10.1186/s12879-024-09455-1 (2024). PMC11145797 38831406

[47] Lebeau, A. et al. HPV infection alters vaginal microbiome through down-regulating host mucosal innate peptides used by Lactobacilli as amino acid sources. *Nat. Commun.* **13**, 1076 10.1038/s41467-022-28724-8 (2022). PMC8885657 35228537

[48] Usyk, M. et al. Cervicovaginal microbiome and natural history of HPV in a longitudinal study. *PLOS Pathog.* **16**, e1008376 10.1371/journal.ppat.1008376 (2020). PMC7098574 32214382

[49] Brotman, R. M. et al. Interplay between the temporal dynamics of the vaginal microbiota and human papillomavirus detection. *J. Infect. Dis.* **210**, 1723–1733 10.1093/infdis/jiu330 (2014). PMC4296189 24943724

[50] Stosic, M. et al. Inferred functional microbiome features are associated with clinical trajectories of low-grade cervical lesions. *BMC Microbiol.* **26**, 802 10.1186/s12866-026-05648-7 (2026). PMC13570522 42732072

[51] Wang, T. et al. Human papillomavirus molecular prevalence in south China and the impact on vaginal microbiome of unvaccinated women. *mSystems* **9**, e00738–24 10.1128/msystems.00738-24 (2024). PMC11407003 39120153

[52] Qingqing, B. et al. Cervicovaginal microbiota dysbiosis correlates with HPV persistent infection. *Microb. Pathogenesis* **152**, 104617 10.1016/j.micpath.2020.104617 (2021). 33207260

[53] Myeong, J. et al. Microbial metabolites control self-renewal and precancerous progression of human cervical stem cells. *Nat. Commun.* **16**, 2327 10.1038/s41467-025-57323-6 (2025). PMC11890575 40057497

[54] Mirzaei, R. et al. Microbiota metabolites in the female reproductive system: Focused on the short-chain fatty acids. *Heliyon* **9**, e14562 10.1016/j.heliyon.2023.e14562 (2023). PMC10031489 36967966

[55] Schwecht, I., Nazli, A., Gill, B. & Kaushic, C. Lactic acid enhances vaginal epithelial barrier integrity and ameliorates inflammatory effects of dysbiotic short chain fatty acids and HIV-1. *Sci. Rep.* **13**, 20065 10.1038/s41598-023-47172-y (2023). PMC10654711 37973920

[56] Usyk, M. et al. molBV reveals immune landscape of bacterial vaginosis and predicts human papillomavirus infection natural history. *Nat. Commun.* **13**, 233 10.1038/s41467-021-27628-3 (2022). PMC8752746 35017496

[57] Liu, H., Liang, H., Li, D., Wang, M. & Li, Y. Association of cervical dysbacteriosis, HPV oncogene expression, and cervical lesion progression. *Microbiol. Spectr.* **10**, e00151–22 10.1128/spectrum.00151-22 (2022). PMC9602310 36036584

[58] Molina, M. A. & Dai, W. Microbiome–metabolome crosstalk in HPV pathogenesis: from ecosystem dynamics to translational biomarkers. *Comput. Struct. Biotechnol. J.* **35**, 0158 10.34133/csbj.0158 (2026). PMC13329037 42404061

[59] Peng, K. et al. Lactobacillus crispatus-derived nCEV vesicles promote cutaneous wound healing and inhibit HPV16 infection. *mSystems* **10**, e00683–25 10.1128/msystems.00683-25 (2025). PMC12455947 40827924

[60] Bridy, P. et al. Human papillomavirus 16 mitigates Sneathia vaginalis-induced damage to cervical keratinocytes. *mSphere* **10**, e00152–25 10.1128/msphere.00152-25 (2025). PMC12306162 40590527

[61] Jung, D.-R. et al. Metagenomic insight into the vaginal microbiome in women infected with HPV 16 and 18. *npj Biofilms Microbiomes* **11**, 105 10.1038/s41522-025-00747-1 (2025). PMC12162855 40506497

[62] Jimenez, N. R., Mancilla, V., Łaniewski, P. & Herbst-Kralovetz, M. M. Immunometabolic contributions of Atopobiaceae family members in human papillomavirus infection, cervical dysplasia, and cancer. *J. Infect. Dis.* **232**, 767–778 10.1093/infdis/jiae533 (2025). PMC13267887 39485269

[63] Cheng, L. et al. Vaginal microbiota and human papillomavirus infection among young Swedish women. *npj Biofilms Microbiomes* **6**, 39 10.1038/s41522-020-00146-8 (2020). PMC7552401 33046723

[64] Cheng, L., Yan, C., Yang, Y., Hong, F. & Du, J. Exploring the clinical signatures of cervical dysplasia patients and their association with vaginal microbiota. *Cancer Med.* **13**, e70440 10.1002/cam4.70440 (2024). PMC11622153 39641435

[65] Passarelli, G. V., Whang, S. N., Gilbert, N. M. & Hu, J. The vaginal microbiome, papillomavirus infection, and cervical cancer: established associations in search of model systems and mechanistic answers. *mBio* **17**, e02677–25 10.1128/mbio.02677-25 (2026). PMC12977512 41665359

[66] Schuppe Koistinen, I. et al. Vaginal dysbiosis and the potential of vaginal microbiome-directed therapeutics. *Front. Microbiomes* **3**, 363089 (2024). 10.3389/frmbi.2024.1363089 PMC12993552 41853538

[67] van der Veer, C. et al. Comparative genomics of human Lactobacillus crispatus isolates reveals genes for glycosylation and glycogen degradation: implications for in vivo dominance of the vaginal microbiota. *Microbiome* **7**, 49 10.1186/s40168-019-0667-9 (2019). PMC6441167 30925932

[68] Sungur, T., Aslim, B., Karaaslan, C. & Aktas, B. Impact of Exopolysaccharides (EPSs) of Lactobacillus gasseri strains isolated from human vagina on cervical tumor cells (HeLa). *Anaerobe* **47**, 137–144 10.1016/j.anaerobe.2017.05.013 (2017). 28554813

[69] Chetwin, E. et al. Antimicrobial and inflammatory properties of South African clinical Lactobacillus isolates and vaginal probiotics. *Sci. Rep.* **9**, 1917 10.1038/s41598-018-38253-4 (2019). PMC6374385 30760770

[70] Pan, M., Hidalgo-Cantabrana, C., Goh, Y. J., Sanozky-Dawes, R. & Barrangou, R. Comparative analysis of Lactobacillus gasseri and Lactobacillus crispatus isolated from human urogenital and gastrointestinal tracts. *Front. Microbiol.* **10**, 3146 (2020). 10.3389/fmicb.2019.03146 PMC6988505 32038579

[71] Mancabelli, L. et al. Comparative genome analyses of Lactobacillus crispatus isolates from different ecological niches reveal an adaptation of this species to the human vaginal environment. *Appl. Environ. Microbiol.* **87**, e02899–20 10.1128/AEM.02899-20 (2021). PMC8091109 33579685

[72] Mejía-Caballero, A. & Marco, M. L. Lactobacilli biology, applications and host interactions. *Nat. Rev. Microbiol.* 10.1038/s41579-025-01205-7 (2025). 40702328

[73] Donders, G. et al. The use of 3 selected lactobacillary strains in vaginal probiotic gel for the treatment of acute Candida vaginitis: a proof-of-concept study. *Eur. J. Clin. Microbiol. Infect. Dis.* **39**, 1551–1558 10.1007/s10096-020-03868-x (2020). 32356027

[74] Spacova, I. et al. Multifactorial inhibition of Candida albicans by combinations of lactobacilli and probiotic Saccharomyces cerevisiae CNCM I-3856. *Sci. Rep.* **14**, 9365 10.1038/s41598-024-59869-9 (2024). PMC11039719 38654026

[75] Allonsius, C. N. et al. Inhibition of Candida albicans morphogenesis by chitinase from Lactobacillus rhamnosus GG. *Sci. Rep.* **9**, 2900 10.1038/s41598-019-39625-0 (2019). PMC6393446 30814593

[76] Lyu, J. et al. From whole genomes to probiotic candidates: a study of potential lactobacilli strains selection for vaginitis treatment. *Heliyon* **10** 10.1016/j.heliyon.2024.e30495(2024). PMC11098787 38765070

[77] Lee, C. Y. et al. Quantitative modeling predicts mechanistic links between pre-treatment microbiome composition and metronidazole efficacy in bacterial vaginosis. *Nat. Commun.* **11**, 6147 10.1038/s41467-020-19880-w (2020). PMC7708644 33262350

[78] Verwijs, M. C., Agaba, S. K., Darby, A. C. & van de Wijgert, J. H. H. M. Impact of oral metronidazole treatment on the vaginal microbiota and correlates of treatment failure. *Am. J. Obstet. Gynecol.* **222**, 157.e151–157.e113 10.1016/j.ajog.2019.08.008 (2020). PMC6995998 31404542

[79] Kalia, V. C., Patel, S. K. S. & Lee, J.-K. Bacterial biofilm inhibitors: an overview. *Ecotoxicol. Environ. Saf.* **264**, 115389 10.1016/j.ecoenv.2023.115389 (2023). 37634478

[80] Wang, S. et al. Strategy to combat biofilms: a focus on biofilm dispersal enzymes. *npj Biofilms Microbiomes* **9**, 63 10.1038/s41522-023-00427-y (2023). PMC10485009 37679355

[81] Molina, M. A., Biswas, S., Núñez-Samudio, V. & Landires, I. Targeting Megasphaera species to promote cervicovaginal health. *Trends Microbiol.* 10.1016/j.tim.2024.04.015 (2024). 38777699

[82] Anton, L. et al. Gardnerella vaginalis alters cervicovaginal epithelial cell function through microbe-specific immune responses. *Microbiome* **10**, 119 10.1186/s40168-022-01317-9 (2022). PMC9351251 35922830

[83] Atassi, F., Brassart, D., Grob, P., Graf, F. & Servin, A. L. Lactobacillus strains isolated from the vaginal microbiota of healthy women inhibit Prevotella bivia and Gardnerella vaginalis in coculture and cell culture. *FEMS Immunol. Med. Microbiol.* **48**, 424–432 10.1111/j.1574-695X.2006.00162.x (2006). 17059467

[84] Zhang, Q. et al. Inhibitory effect of Lactobacillus gasseri CCFM1201 on Gardnerella vaginalis in mice with bacterial vaginosis. *Arch. Microbiol.* **204**, 315 10.1007/s00203-022-02896-9 (2022). 35546374

[85] Gao, Q. et al. Lactobacillus gasseri LGV03 isolated from the cervico-vagina of HPV-cleared women modulates epithelial innate immune responses and suppresses the growth of HPV-positive human cervical cancer cells. *Transl. Oncol.* **35**, 101714 10.1016/j.tranon.2023.101714 (2023). PMC10366645 37331103

[86] Navarro, S., Abla, H., Colmer-Hamood, J. A., Ventolini, G. & Hamood, A. N. Under conditions closely mimicking vaginal fluid, Lactobacillus jensenii strain 62B produces a bacteriocin-like inhibitory substance that targets and eliminates Gardnerella species. *Microbiology* **169** 10.1099/mic.0.001409(2023). PMC10710834 37909284

[87] Zeng, Z., Zuo, F. & Marcotte, H. Putative adhesion factors in vaginal Lactobacillus gasseri DSM 14869: functional characterization. *Appl. Environ. Microbiol.* **85**, e00800–19 10.1128/AEM.00800-19 (2019). PMC6752014 31420338

[88] Brusselaers, N., Shrestha, S., van de Wijgert, J. & Verstraelen, H. Vaginal dysbiosis and the risk of human papillomavirus and cervical cancer: systematic review and meta-analysis. *Am. J. Obstet. Gynecol.* **221**, 9–18.e18 10.1016/j.ajog.2018.12.011 (2019). 30550767

[89] Yang, Z. et al. Vaginal microbiota and personal risk factors associated with HPV status conversion—A new approach to reduce the risk of cervical cancer? *PLoS ONE* **17**, e0270521 10.1371/journal.pone.0270521 (2022). PMC9362946 35944043

[90] Vodstrcil, L. A., Muzny, C. A., Plummer, E. L., Sobel, J. D. & Bradshaw, C. S. Bacterial vaginosis: drivers of recurrence and challenges and opportunities in partner treatment. *BMC Med.* **19**, 194 10.1186/s12916-021-02077-3 (2021). PMC8411528 34470644

[91] Raba, G. et al. Efficacy of dequalinium chloride vs metronidazole for the treatment of bacterial vaginosis: a randomized clinical trial. *JAMA Netw. Open* **7**, e248661 10.1001/jamanetworkopen.2024.8661 (2024). PMC11066704 38696172

[92] Potloane, D. et al. VIBRANT: A phase 1 randomized trial of multi-strain vaginal L. crispatus live biotherapeutic products in people with bacterial vaginosis. *Cell Host Microbe* **34**, 751–760.e5 10.1016/j.chom.2026.02.016 (2026). 41856107

[93] Lebeer, S. et al. Selective targeting of skin pathobionts and inflammation with topically applied lactobacilli. *Cell Reports Medicine* **3** 10.1016/j.xcrm.2022.100521(2022). PMC8861818 35243421

[94] De Boeck, I. et al. Randomized, double-blind, placebo-controlled trial of a throat spray with selected Lactobacilli in COVID-19 outpatients. *Microbiol. Spectr.* **10**, e01682–22 10.1128/spectrum.01682-22 (2022). PMC9604152 36154666

[95] Spacova, I. et al. Development of a live biotherapeutic throat spray with lactobacilli targeting respiratory viral infections. *Microb. Biotechnol.* **16**, 99–115 10.1111/1751-7915.14189 (2023). PMC9803329 36468246

[96] Paetzold, B. et al. Skin microbiome modulation induced by probiotic solutions. *Microbiome* **7**, 95 10.1186/s40168-019-0709-3 (2019). PMC6591853 31234928

[97] Dellino, M. et al. Lactobacillus crispatus M247 oral administration: Is it really an effective strategy in the management of papillomavirus-infected women? *Infect. Agents Cancer* **17**, 53 10.1186/s13027-022-00465-9 (2022). PMC9587645 36271433

[98] Lavitola, G., Della Corte, L., De Rosa, N., Nappi, C. & Bifulco, G. Effects on vaginal microbiota restoration and cervical epithelialization in positive HPV patients undergoing vaginal treatment with carboxy-methyl-beta-glucan. *BioMed. Res. Int.* **2020**, 5476389 10.1155/2020/5476389 (2020). PMC7201736 32420349

[99] Serrano, L. et al. Efficacy of a coriolus versicolor–based vaginal gel in women with human papillomavirus–dependent cervical lesions: the PALOMA study. *J. Low Genit. Tract. Dis.* **25**, 130–136 (2021). 10.1097/LGT.0000000000000596 PMC7984756 33746195

[100] Jenkins, D. J. et al. Bacterial amylases enable glycogen degradation by the vaginal microbiome. *Nat. Microbiol.* **8**, 1641–1652 10.1038/s41564-023-01447-2 (2023). PMC10465358 37563289

[101] Talbert, J. A., Spicer, S. K., Manning, S. D., Gaddy, J. A. & Townsend, S. D. Human milk oligosaccharides mediate the host-microbe interface in a model vaginal community. *ACS Infect. Dis.* **11**, 1729–1740 10.1021/acsinfecdis.5c00295 (2025). PMC12172043 40459557

[102] Tidbury, F. et al. Effectiveness and tolerability of lactic acid vaginal gel compared to oral metronidazole in the treatment of acute symptomatic bacterial vaginosis: a multicenter, randomized-controlled, head-to-head pilot study. *BMC Women’s. Health* **25**, 7 10.1186/s12905-024-03513-1 (2025). PMC11702144 39762879

[103] Rouanet, A. et al. Live biotherapeutic products, a road map for safety assessment. *Front. Med.* **7**, 237 10.3389/fmed.2020.00237 (2020). PMC7319051 32637416

[104] Gilbert, J. A. et al. Clinical translation of microbiome research. *Nat. Med.* **31**, 1099–1113 10.1038/s41591-025-03615-9 (2025). 40217076

[105] Pandey, K., Gosai, H. & Patel, R. B. An updated review on advantages, disadvantages and uncertainties associated with probiotics. *J. Dairy Res.* **92**, 329–344 10.1017/S0022029925101155 (2025). 41158065

[106] Wei, F., Georges, D., Man, I., Baussano, I. & Clifford, G. M. Causal attribution of human papillomavirus genotypes to invasive cervical cancer worldwide: a systematic analysis of the global literature. *Lancet* **404**, 435–444 10.1016/S0140-6736(24)01097-3 (2024). 39097395

[107] Molina, M. A., Steenbergen, R. D. M., Pumpe, A., Kenyon, A. N. & Melchers, W. J. G. HPV integration and cervical cancer: a failed evolutionary viral trait. *Trends Mol. Med.* **30**, 890–902 10.1016/j.molmed.2024.05.009 (2024). 38853085

[108] Perkins, R. B. et al. 2019 ASCCP risk-based management consensus guidelines: updates through 2023. *J. Low Genit. Tract. Dis.* **28**, 3–6 (2024). 10.1097/LGT.0000000000000788 PMC10755815 38117563

[109] Cibula, D. et al. ESGO/ESTRO/ESP guidelines for the management of patients with cervical cancer – Update 2023*. *Int. J. Gynecol. Cancer* **33**, 649–666 10.1136/ijgc-2023-004429 (2023). PMC10176411 37127326

