# Supplementary material for: Towards a sequential microbiome therapy for HPV-associated cervicovaginal dysbiosis
Source: Commun Biol. 2026 Oct 29;9:1422. doi: 10.1038/s42003-026-11208-6 (PMC13720077; doi:10.1038/s42003-026-11208-6)
Supplement: Supplementary file 1 [file 42003_2026_11208_MOESM1_ESM.pdf]

nature portfolio

Peer Review File

Towards a sequential microbiome therapy for HPV-associated cervicovaginal dysbiosis

Corresponding Author: Dr Mariano Molina

This file contains all reviewer reports in order by version, followed by all author rebuttals in order by version.

Version 0:

Reviewer comments:

Reviewer #1

(Remarks to the Author)
This perspective manuscript by Molina and colleagues introduces Sequential Microbiome Therapy (SMT), a novel framework designed to counteract high-risk human papillomavirus (hrHPV) persistence by restoring the cervicovaginal microbiome. The authors argue that hrHPV is intrinsically linked to microbial instability, specifically the transition from healthy Lactobacillus-dominant states (CST I, II, V) to diverse, anaerobic communities (CST IV) or L. iners-dominated states (CST III). SMT is structured as a two-phase strategy: the first phase utilizes biofilm-disrupting agents, specific inhibitors (like oleic acid), or narrow-spectrum antimicrobials to "reset" a dysbiotic environment. The second phase involves the strategic reintroduction of functionally selected Lactobacillus strains—specifically L. crispatus, L. gasseri, or L. jensenii—chosen for their high acidification capacity and ability to outcompete pathogens and inhibit viral entry. This perspective highlights that traditional monotherapies, such as probiotics or antibiotics, often fail due to the cervical microbiome's dynamic nature and the failure of non-vaginal strains to colonize effectively. By leveraging strain-level specificity and targeting the bidirectional feedback loop between hrHPV and the microbiome, SMT aims to create a stable, long-lasting ecological barrier against viral persistence and neoplastic progression. The authors emphasize that this approach is donor-independent, scalable, and could be implemented via self-administered gels or sprays, making it particularly viable for low-resource settings where cervical cancer remains a primary health threat.

As a perspective article, this proposal is visionary and interesting. While the manuscript cites several 2024-2025 papers, its completeness depends on addressing findings from recent high-impact trials in the field that are not fully cited. The manuscript lacks a comprehensive presentation of the current state of the art in the cervicovaginal microbiome-modulating interventions field. A dedicated section reviewing microbiome-targeted therapeutic approaches for hrHPV infection and cervical disease is necessary to properly contextualize the novelty and advantages of the authors' strategy. Providing this background will substantially strengthen the manuscript by situating the proposed therapy within the global research landscape and substantiating the claimed advantages over current approaches. Please see some points below:
1. The manuscript mentions L. crispatus colonization but should more explicitly cite recent longitudinal data from trials of LACTIN-V (L. crispatus CTV-05). Recent studies (e.g., Hemmerling A et al., Sex Transmit Dis 2024; Armstrong E et al., Microbiome 2024) have explored its efficacy in preventing BV recurrence, which serves as the primary proof-of-concept for the "Phase 2" of SMT.
2. Within the domain of host-microbe metabolic crosstalk, the manuscript discusses lactic acid but could also cite recent work on short-chain fatty acids (SCFAs) and their specific role in modulating T-cell responses in the cervical transformation zone, which are important in cancer prevention and treatment. Examples include Mafe and Büsselberg, Biomolecules 2025; Kim and Yang, Nutrients 2024).

Some unexpected limitations may also arise during the SMT strategy and the authors can propose additional adjustments to their strategy to tackle them. For example, there may be some colonization resistance due to resilient biofilms that "Phase 1" may not fully clear, leading to the rejection of introduced Lactobacillus strains. In such cases, the incorporation of metabolic priming (e.g., providing prebiotics like glycogen or specific human milk oligosaccharides) alongside the Lactobacillus introduction may give the therapeutic strains a metabolic "head start". Also, the manuscript mentions the menstrual cycle, but does not detail how fluctuating estrogen levels might disrupt the SMT phases. In this case, timing the "Phase 2" introduction to the follicular phase (when estrogen levels are rising) may be a good option, as this condition naturally supports Lactobacillus dominance via increased glycogen deposition.

Minor comments:
In Figure 1, the arrow regarding CVM stability should point from Lactobacillus strains to CVM stability.

In Figure 2.1a, some bacteria taxa that should be inhibited are missing. In Figure 2.1b, cysteine inhibitor should be listed with the other L. iners community inhibitors (BDA, oleic acid,…) in the left side. BDA and other abbreviations in the figure should be explained in the figure captions.

In line 166: "This is accomplished by administering a second treatment containing L. crispatus, L. gasseri, or L. jensenii strains that exert a strong adherence to cervicovaginal epithelial cells and/or inhibit hrHPV entry into host cells (Figure 2.2)." References here (56-58) do not support the statement that Lactobacillus strains inhibit hrHPV entry into host cells. I suggest providing additional evidence or rephrasing the claim.

Reviewer #2

(Remarks to the Author)

This perspective, much reading like a motivation for a research proposal, presents an interesting and novel concept to be tested in practise. Few comments to address:

-The causality of polymicrobial CVM and persistent HPV infection has not been proven and this must be clearly stated. As we still have no effective cure with long lasting results for BV, it is kind of early to propose a cure for another infection by curing BV.

- The authors must add discussion about the possible application routes for the stages 1 and 2 and acknowledge that at least in Europe, the proposed SMT approach falls under a medicinal product category in regulatory terms. This mean that for human trials, any Lactobacillus-product to be applied vaginally for the treatment of HPV-associated cervicovaginal dysbiosis must be manufactured as GMP grade and registered as a Live Biotherapeutic Product under biological medicines. This is prohibitively expensive. Hence, the term cost-effective in the abstract must be removed or explained to be conditional.

-Figure 1. Is not a proper Venn diagram. What happens in the overlapping areas?

Reviewer #3

(Remarks to the Author)
I co-reviewed this manuscript with one of the reviewers who provided the listed reports. This is part of the Communications Biology initiative to facilitate training in peer review and to provide appropriate recognition for Early Career Researchers who co-review manuscripts.

Reviewer #4

(Remarks to the Author)
This generally well written but under referenced paper reviews the concept of Sequential Microbiome Therapy (SMT) aimed at improving cervicovaginal health and potentially helping to clear high-risk HPV (hrHPV) infection. This review considered dynamic factors like age and menstrual cycle, which are often overlooked in current treatments but failed to fully discuss the impact of ethnicity on what would be a 'normal' vaginal microbiome. In fact, a more careful discussion of the eubiotic community common in those ethnicities that show greatest susceptibility to hrHPV infection and consequence would greatly enhance this review and likely offer stronger support for SMT. As presented, the review offers substantial conjecture as to which approach/approaches for phase 1 but fails to offer a priority approach for study. Phase 2 provides an implication that a one size fits all is a best approach but this is under-supported and should be better addressed using more of the meta-analyses in the literature. In general, the idea that SMT could lead to long-lasting microbial stability to support hrHPV clearance is not novel per se but does help focus future research efforts to test if a Lactobacillus-dominated probiotic could offer measurable impacts against HPV. Overall there is opportunity for some reorganization to enhance readability and reduce redundancy in the content.

Abstract and General Terminology
• In the abstract (lines 15-16), terms like "pre-cancer" and "cancer" might be vague. Using more precise terms like "Cervical Intraepithelial Neoplasia" (CIN), that was clearly described in your introduction would enhance the asbtract.

Introduction
• Line 32 is unnecessary.
• Lines 33-35: The current phrasing minimizes the importance of this issue. It would be beneficial to emphasize that HPV causes over 90% of all cervical cancers, with the remainder attributed to in utero exposure to teratogens and genetic diseases. Cervical cancer is the most common gynecologic malignancy globally and is often diagnosed in later stages where surgery is no longer considered curative. There are many recent reviews that could be cited for the reader's education.
• Consider mentioning SMT as an adjunct to current cervical dysplasia treatment guidelines.

• In the introduction (lines 44-45), it is noted that SMT will be compared to existing therapeutic modalities, specifically Vaginal Microbiome Transplantation (VMT), standalone probiotic interventions, and antibiotics. While SMT's advantage over antibiotics (e.g., metronidazole can clear detrimental microbial communities but often at the expense of beneficial ones) is explained later, more detail would be beneficial for VMT and standalone probiotics. For instance, beyond SMT being donor-independent, expansion of other key advantages or disadvantages when comparing SMT to VMT would be helpful. A clearer comparison with standalone probiotic use would also strengthen this section. Similarly, the practice of douching or other hygiene practices is associated with greater diversity in the community but is not discussed in the review.

Add a HPV and Cervical Dysplasia Overview
• A specific section, similar to the "Cervical microbiome section," that provides a brief overview of HPV and cervical dysplasia would be of value. Within such an overview, please use clinically appropriate terminology when discussing cervical dysplasia. Clarify definitions of "early" and "intermediate" disease—is this based on cytology (e.g., LSIL, ASCUS, HSIL) or biopsy-proven pathology (e.g., CIN 1/2/3)? Clinically, CIN 1 has a less than 1% chance of progressing to cancer and often regresses spontaneously. CIN 2 and 3 have significantly higher rates of progression to cervical cancer, and this is typically where Ob-Gyns initiate treatment, with some exceptions based on HPV genotype and patient medical history.
• Please reference the ASCCP guidelines or their European equivalent for how different cytology results and CIN 1/2/3 are managed.
• The sentence contained in lines 186-190, which discusses HPV, feels out of place and should be introduced earlier in the paper, ideally within this new HPV overview.
• When discussing high-risk HPV (hrHPV), further clarity on which hrHPV strains are included is needed. This is important because:
o hrHPV strains are not universally distributed. While HPV 16/18 are prevalent globally and highly mutagenic, other hrHPV strains (e.g., hrHPV 52 and 58 in Central and South America) have higher prevalence and rates of cervical dysplasia/cancer in different regions.
o With increasing vaccination rates, clinicians are observing a decrease in HPV 16/18-related disease and an increasing prevalence of non-16/18 hrHPV-related precancerous and malignant lesions.

Cervicovaginal Microbiome Dynamics
• The sentences in lines 79-82 are a bit awkward and require adjustment.
• Please be more specific about which Lactobacillus species corresponds to each Cervicovaginal State (CST) grouping. In fact the more recently updated CST or other systems are noted but not connected well to the review. Several arguments are made (e.g. lines 171, 178, 191, etc) but no solid conclusion drawn about what strains/species would be prioritized in SMT for phase 1 and 2. L. gasseri is highlighted (lines 171 -173) but potentially under-referenced. If multiple species/strains are to be included some mention of study of their synergism (or at least lack of competition) should be included.
• For changes in the CVM, please clarify the timeframe over which these changes are observed (e.g., days, weeks, months, years). If changes occur over months to years, please additionally clarify how ‘donor-dependent’ (line 197) or the personalize approach would be applied to address the effects of hrHPV and expected changes from the menopausal transition, which can begin as early as the late 30s. Would CVM sampling over time be a critical component or is SMT based on a single snapshot before being initiated? This also seems the proper section to address directed changes in hygiene as part of SMT plans.
• The sentence contained in lines 227 to 229 should be adjusted and moved into this section, as it appears to relate to microbiome dynamics.

The Approach of Sequential Microbiome Therapy (SMT)
• Line 135 on indicates the bacteria that need to be inhibited leading to “disrupting these virus-bacterium feedback loops is therefore a critical goal of the first stage of SMT” but does not then declare the priority approach. The authors posit “top strains” of Lactobacilli spp that lower vaginal pH but should then provide a table or review of the literature for those published strains and their properties if that is their conclusion for phase 2 approach. Others have reported different bacteria as disrupting the virus-bacteria connection but these are not noted. Figure 2 also seems to suggest multiple approaches for phase 1 consistent with a personalized medicine approach but the text fails to convey the specifics. Is the plan to offer oral/vaginal application of the 4 component blend shown in fig 2 for each condition? Are the Lactobacilli in phase 1 those used in phase 2? Again the lack of detail on the Lactobacilli spp reduces the advancement of the field ore refinements/suggestions for best study plans with this review.
• The sentence contained in lines 184-186 should be moved to an earlier point within this section.
• Please clarify if your approach is able to differentiate between the regression of HPV infection and true clearance of the virus.
• Provide more detail on how this therapy is performed. Is it an oral or vaginal medication? Can it be administered at home or in a clinic setting, or will it require laboratory testing in advance?

Advantages and Limitations
• For the preclinical data discussed in lines 206-209, please clarify if regions other than Africa and North America were included in the analysis. Additionally, please specify which country in North America the samples were obtained from. Ultimately a commentary on SMT application in distinct populations would be of value.
• Beyond the need for further testing in other clinical settings, please include additional limitations. For example, clarify if SMT would be useful for "late" HPV disease.
Additional references that should be included or at least noted:
1) https://journals.asm.org/doi/10.1128/msystems.00017-22
2) https://journals.asm.org/doi/10.1128/mbio.02677-25?utm_source=TOC&utm_medium=ealert&utm_campaign=TOC_v17_i3&ref=16e4f3c1-40d6-4d96-804e-6cf3d92cfda5

Figures and Acronyms
• For Figure 1, it would be helpful to include the specific names of the Lactobacillus strains mentioned.
• For Figure 2, "BDA" is not defined in either the figure or the text (biofilm-disrupting agent?).

Reviewer #5

(Remarks to the Author)
I co-reviewed this manuscript with one of the reviewers who provided the listed reports. This is part of the Communications Biology initiative to facilitate training in peer review and to provide appropriate recognition for Early Career Researchers who co-review manuscripts.

Version 1:

Reviewer comments:

Reviewer #1

(Remarks to the Author)
The authors have addressed and complied with every single point raised by myself and the four additional Reviewers that assessed their work in a satisfactory fashion. Therefore, I recommend the acceptance of this revised manuscript in its current form.

Reviewer #2

(Remarks to the Author)
The authors have addressed the points raised and I have no further requests, except to replace the word "may" with "drastically" in the following sentence referring to pharma-grade manufacturing cost in lines 318-318 : " These requirements may increase development costs and.."

Reviewer #5

(Remarks to the Author)
I co-reviewed this manuscript with one of the reviewers who provided the listed reports. This is part of the Communications Biology initiative to facilitate training in peer review and to provide appropriate recognition for Early Career Researchers who co-review manuscripts.

Reviewer #6

(Remarks to the Author)
As a newly-added reviewer after the first round of review, I think the authors in general address the comments very well. I only have a few comments on the authors' response to the second comment of reviewer #1, which is about the "Phase 1" of the SMT on colonization resistance.

In the response the authors expanded the discussion of potential barriers to successful SMT phase 1 implementation, including biofilm colonization resistance and possible supplements of glycogen or milk oligosaccharides. One thing that I am not sure about the phase 1, however, is the agent that is used to inhibit the potential detrimental bacteria. In Figure 1 I can see that there are compounds such as BDA, Cysteine inhibitor, antibiosis, and live bacteria such as Lactobacillus strains. Putting compounds and bacterial strains together, however, may be a bit misleading. While the compounds are known to inhibit bacteria, the effect of the introduction of Lactobacillus strains is not so certain. Yes I know that the authors discuss some of the potential effects of the Lactobacillus in Line 221-229; but the introduction of Lactobacillus strains in Phase 1 almost feels like Phase 2 is not necessary at all. Please carefully think about whether it is really necessary to put Lactobacillus strains in Phase 1 for disruption purpose.

There is one more thing that may need the authors' attention: the potential risk of probiotics. As was discussed in Pandey 2025 (doi:10.1017/S0022029925101155), there are potential risks of administered antibiotics such as horizontal gene transfer, antimicrobial resistance genes, or even infectious diseases caused by the probiotics. Although the introduction of Lactobacillus species into the vaginal environment is not conventional "probiotics", broadly speaking it still roughly falls into this definition. Some discussion would be very helpful in the control of potential risks.

Open Access This Peer Review File is licensed under a Creative Commons Attribution 4.0 International License, which permits use, sharing, adaptation, distribution and reproduction in any medium or format, as long as you give appropriate credit to the original author(s) and the source, provide a link to the Creative Commons license, and indicate if changes were made.
In cases where reviewers are anonymous, credit should be given to 'Anonymous Referee' and the source.
The images or other third party material in this Peer Review File are included in the article's Creative Commons license, unless indicated otherwise in a credit line to the material. If material is not included in the article's Creative Commons license and your intended use is not permitted by statutory regulation or exceeds the permitted use, you will need to obtain permission directly from the copyright holder.
To view a copy of this license, visit https://creativecommons.org/licenses/by/4.0/

Reviewers' comments:

Reviewer #1 (Remarks to the Author):

This perspective manuscript by Molina and colleagues introduces Sequential Microbiome Therapy (SMT), a novel framework designed to counteract high-risk human papillomavirus (hrHPV) persistence by restoring the cervicovaginal microbiome. The authors argue that hrHPV is intrinsically linked to microbial instability, specifically the transition from healthy Lactobacillus-dominant states (CST I, II, V) to diverse, anaerobic communities (CST IV) or L. iners-dominated states (CST III). SMT is structured as a two-phase strategy: the first phase utilizes biofilm-disrupting agents, specific inhibitors (like oleic acid), or narrow-spectrum antimicrobials to "reset" a dysbiotic environment. The second phase involves the strategic reintroduction of functionally selected Lactobacillus strains—specifically L. crispatus, L. gasseri, or L. jensenii—chosen for their high acidification capacity and ability to outcompete pathogens and inhibit viral entry. This perspective highlights that traditional monotherapies, such as probiotics or antibiotics, often fail due to the cervical microbiome's dynamic nature and the failure of non-vaginal strains to colonize effectively. By leveraging strain-level specificity and targeting the bidirectional feedback loop between hrHPV and the microbiome, SMT aims to create a stable, long-lasting ecological barrier against viral persistence and neoplastic progression. The authors emphasize that this approach is donor-independent, scalable, and could be implemented via self-administered gels or sprays, making it particularly viable for low-resource settings where cervical cancer remains a primary health threat.

As a perspective article, this proposal is visionary and interesting. While the manuscript cites several 2024-2025 papers, its completeness depends on addressing findings from recent high-impact trials in the field that are not fully cited. The manuscript lacks a comprehensive presentation of the current state of the art in the cervicovaginal microbiome-modulating interventions field. A dedicated section reviewing microbiome-targeted therapeutic approaches for hrHPV infection and cervical disease is necessary to properly contextualize the novelty and advantages of the authors' strategy. Providing this background will substantially strengthen the manuscript by situating the proposed therapy within the global research landscape and substantiating the claimed advantages over current approaches. Please see some points below:
R: We thank the reviewer for this valuable suggestion. In response, we have added a new section reviewing current microbiome-directed interventions, including antibiotics, probiotics, LBPs, VMT, and emerging biofilm- and metabolite-targeted approaches (Lines 51-83, Table 1). We have also incorporated recent studies and expanded the discussion to contextualize SMT within the current therapeutic landscape better.

1. The manuscript mentions L. crispatus colonization but should more explicitly cite recent longitudinal data from trials of LACTIN-V (L. crispatus CTV-05). Recent studies (e.g., Hemmerling A et al., Sex Transmit Dis 2024; Armstrong E et al., Microbiome 2024) have explored its efficacy in preventing BV recurrence, which serves as the primary proof-of-concept for the "Phase 2" of SMT.
R: We appreciate the reviewer for highlighting these important studies. Accordingly, we expanded the discussion of LACTIN-V and incorporated the recommended references, together with additional recent longitudinal data on *L. crispatus* engraftment and microbiome stabilization (Lines 62-66). We agree that these studies provide important support for the restoration phase of the proposed SMT framework.

2. Within the domain of host-microbe metabolic crosstalk, the manuscript discusses lactic acid but could also cite recent work on short-chain fatty acids (SCFAs) and their specific role in modulating T-cell responses in the cervical transformation zone, which are important in cancer prevention and treatment. Examples include Mafe and Büsselberg, Biomolecules 2025; Kim and Yang, Nutrients 2024).
R: The reviewer raises an important point regarding host-microbe metabolic interactions. In response, we have briefly expanded the discussion of microbial metabolites by incorporating recent literature on SCFAs and their roles in immune modulation, epithelial homeostasis, and the cervicovaginal microenvironment (Lines 137-140).

Some unexpected limitations may also arise during the SMT strategy and the authors can propose additional adjustments to their strategy to tackle them. For example, there may be some colonization resistance due to resilient biofilms that “Phase 1” may not fully clear, leading to the rejection of introduced Lactobacillus strains. In such cases, the incorporation of metabolic priming (e.g., providing prebiotics like glycogen or specific human milk oligosaccharides) alongside the Lactobacillus introduction may give the therapeutic strains a metabolic “head start”. Also, the manuscript mentions the menstrual cycle, but does not detail how fluctuating estrogen levels might disrupt the SMT phases. In this case, timing the "Phase 2" introduction to the follicular phase (when estrogen levels are rising) may be a good option, as this condition naturally supports Lactobacillus dominance via increased glycogen deposition.
R: We appreciate this insightful comment. In response, we expanded the discussion to address potential barriers to successful SMT implementation, including residual biofilm-mediated colonization resistance and the possibility of enhancing *Lactobacillus* engraftment through metabolic priming approaches such as glycogen supplementation and human milk oligosaccharides (Lines 331-334). We also expanded the discussion of menstrual cycle dynamics and how this may improve engraftment efficiency and long-term microbiome stability (Lines 113-116).

Minor comments:
In Figure 1, the arrow regarding CVM stability should point from Lactobacillus strains to CVM stability.
R: We have revised Figure 1 accordingly and corrected the direction of the arrow to indicate the relationship between *Lactobacillus* strains and CVM stability more accurately.

In Figure 2.1a, some bacteria taxa that should be inhibited are missing. In Figure 2.1b, cysteine inhibitor should be listed with the other L. iners community inhibitors (BDA, oleic acid,…) in the left side. BDA and other abbreviations in the figure should be explained in the figure captions.
R: We appreciate the reviewer’s careful evaluation of the figures. We have revised Figure 2 to include the additional dysbiosis-associated taxa, added cysteine inhibitors alongside the other *L. iners*-targeting interventions in Figure 2.1b, and expanded the figure caption to define BDA and all other abbreviations used in the figure (Lines 240-249).

In line 166: “This is accomplished by administering a second treatment containing L. crispatus, L. gasseri, or L. jensenii strains that exert a strong adherence to cervicovaginal epithelial cells and/or inhibit hrHPV entry into host cells (Figure 2.2).” References here (56-58) do not support the statement that Lactobacillus strains inhibit hrHPV entry into host cells. I suggest providing additional evidence or rephrasing the claim.
R: We thank the reviewer for pointing this out. We have revised the text to more accurately reflect the available evidence (Lines 254-255).

Reviewer #2 (Remarks to the Author):

This perspective, much reading like a motivation for a research proposal, presents an interesting and novel concept to be tested in practise. Few comments to address:

-The causality of polymicrobial CVM and persistent HPV infection has not been proven and this must be clearly stated. As we still have no effective cure with long lasting results for BV, it is kind of early to propose a cure for another infection by curing BV.
R: We agree with the reviewer that causality between cervicovaginal dysbiosis and persistent hrHPV infection has not been conclusively demonstrated. The intention of this *Perspective* is not to imply a causal relationship or propose SMT as a cure for hrHPV infection, but to present a hypothesis-generating framework based on the growing body of evidence linking cervicovaginal microbial composition with hrHPV persistence and cervical disease outcomes. To avoid overinterpretation, we have further clarified this point in the revised manuscript (Lines 121-125).

- The authors must add discussion about the possible application routes for the stages 1 and 2 and acknowledge that at least in Europe, the proposed SMT approach falls under a medicinal product category in regulatory terms. This mean that for human trials, any Lactobacillus-product to be applied vaginally for the treatment of HPV-associated cervicovaginal dysbiosis must be manufactured as GMP grade and registered as a Live Biotherapeutic Product under biological medicines. This is prohibitively expensive. Hence, the term cost-effective in the abstract must be removed or explained to be conditional.
R: We appreciate the reviewer for raising this important point. We have expanded the discussion to address potential routes of administration and the regulatory considerations associated with SMT, including the requirements for LBPs (Lines 336-341). We have also revised the abstract to avoid overstating the potential cost-effectiveness of the approach (Lines 31-32).

-Figure 1. Is not a proper Venn diagram. What happens in the overlapping areas?
R: We thank the reviewer for this observation. Figure 1 has been revised to improve clarity and avoid ambiguity regarding the overlapping regions. The updated figure now more clearly illustrates the relationships among the concepts and their contribution to the proposed SMT framework.

Reviewer #3 (Remarks to the Author):

I co-reviewed this manuscript with one of the reviewers who provided the listed reports. This is part of the Communications Biology initiative to facilitate training in peer review and to provide appropriate recognition for Early Career Researchers who co-review manuscripts.

Reviewer #4 (Remarks to the Author):

This generally well written but under referenced paper reviews the concept of Sequential Microbiome Therapy (SMT) aimed at improving cervicovaginal health and potentially helping to clear high-risk HPV (hrHPV) infection. This review considered dynamic factors like age and menstrual cycle, which are often overlooked in current treatments but failed to fully discuss the impact of ethnicity on what would be a 'normal' vaginal microbiome. In fact, a more careful discussion of the eubiotic community common in those ethnicities that show greatest susceptibility to hrHPV infection and consequence would greatly enhance this review and likely offer stronger support for SMT. As presented, the review offers substantial conjecture as to which approach/approaches for phase 1 but fails to offer a priority approach for study. Phase 2 provides an implication that a one size fits all is a best approach but this is under-supported and should be better addressed using more of the meta-analyses in the literature. In general, the idea that SMT could lead to long-lasting microbial stability to support hrHPV clearance is not novel per se but does help focus future research efforts to test if a Lactobacillus-dominated probiotic could offer measurable impacts against HPV. Overall there is opportunity for some reorganization to enhance readability and reduce redundancy in the content.
R: We appreciate the reviewer's thoughtful assessment and constructive suggestions. In response, we expanded the discussion of population-specific cervicovaginal microbiome compositions and ethnicity-related differences in eubiosis, incorporated additional literature to better support strain selection and microbiome restoration strategies, and clarified the rationale and limitations of the proposed SMT framework (Lines 104-111). We also revised the manuscript to improve organization, reduce redundancy, and enhance overall readability.

Abstract and General Terminology
• In the abstract (lines 15-16), terms like "pre-cancer" and "cancer" might be vague. Using more precise terms like "Cervical Intraepithelial Neoplasia" (CIN), that was clearly described in your introduction would enhance the abstract.
R: We appreciate this suggestion. To improve precision and consistency of terminology throughout the manuscript, we revised the abstract to replace the terms "precancer" with cervical intraepithelial neoplasia (CIN) where appropriate (Line 16, Box 1).

Postal address
Department of Laboratory Medicine
Division of Biomolecular and Cellular Medicine
171 77 Stockholm, Sweden
Org. nummer 202100 2973

Visiting address
Alfred Nobels Allé 8
14152 Huddinge
Stockholm
Sweden

Contact
E-mail: **mariano.molina.beitia@ki.se**

Introduction
• Line 32 is unnecessary.
R: We thank the reviewer for this suggestion. The sentence this line (now Line 37) has been removed from the revised manuscript.

• Lines 33-35: The current phrasing minimizes the importance of this issue. It would be beneficial to emphasize that HPV causes over 90% of all cervical cancers, with the remainder attributed to in utero exposure to teratogens and genetic diseases. Cervical cancer is the most common gynecologic malignancy globally and is often diagnosed in later stages where surgery is no longer considered curative. There are many recent reviews that could be cited for the reader's education.
R: We appreciate the reviewer's comment. In response, we added Box 1 where emphasize the global burden of cervical cancer. We also incorporated additional references to provide a broader context on cervical cancer epidemiology and prevention.

• Consider mentioning SMT as an adjunct to current cervical dysplasia treatment guidelines.
R: We have expanded the discussion to clarify that SMT is envisioned as a complementary strategy that could potentially support existing cervical cancer prevention and management approaches, rather than replace current guideline-recommended interventions for cervical dysplasia (Box 1).

• In the introduction (lines 44-45), it is noted that SMT will be compared to existing therapeutic modalities, specifically Vaginal Microbiome Transplantation (VMT), standalone probiotic interventions, and antibiotics. While SMT's advantage over antibiotics (e.g., metronidazole can clear detrimental microbial communities but often at the expense of beneficial ones) is explained later, more detail would be beneficial for VMT and standalone probiotics. For instance, beyond SMT being donor-independent, expansion of other key advantages or disadvantages when comparing SMT to VMT would be helpful. A clearer comparison with standalone probiotic use would also strengthen this section. Similarly, the practice of douching or other hygiene practices is associated with greater diversity in the community but is not discussed in the review.
R: We appreciate this thoughtful comment. In response, we expanded the discussion of current microbiome-directed interventions to provide a more comprehensive comparison between SMT, VMT, probiotics, and antibiotic-based approaches, including their respective advantages and limitations (Lines 51-83, Table 1). We also added discussion of behavioral and environmental factors, such as vaginal hygiene practices, that may influence cervicovaginal microbiome composition and treatment outcomes (Lines 112-120).

Add a HPV and Cervical Dysplasia Overview
• A specific section, similar to the "Cervical microbiome section," that provides a brief overview of HPV and cervical dysplasia would be of value. Within such an overview, please use clinically appropriate terminology when discussing cervical dysplasia. Clarify definitions of "early" and "intermediate" disease—is this based on cytology (e.g., LSIL, ASCUS, HSIL) or biopsy-proven pathology (e.g., CIN 1/2/3)? Clinically, CIN 1 has a less than 1% chance of progressing to cancer and often regresses spontaneously. CIN 2 and 3 have significantly higher rates of progression to cervical cancer, and this is typically where Ob-Gyns initiate treatment, with some exceptions based on HPV genotype and patient medical history.
R: We appreciate this suggestion. To improve clarity for a broader readership, we expanded these concepts and added Box 1 to explain these terms.

• Please reference the ASCCP guidelines or their European equivalent for how different cytology results and CIN 1/2/3 are managed.
R: We agree with the reviewer. We have added a reference to the cervical cancer management guidelines in Box 1 .

• The sentence contained in lines 186-190, which discusses HPV, feels out of place and should be introduced earlier in the paper, ideally within this new HPV overview.

Postal address
Department of Laboratory Medicine
Division of Biomolecular and Cellular Medicine
171 77 Stockholm, Sweden
Org. nummer 202100 2973

Visiting address
Alfred Nobels Allé 8
14152 Huddinge
Stockholm
Sweden

Contact
E-mail: **mariano.molina.beitia@ki.se**

R: We appreciate this observation. To improve the flow and organization of the manuscript, we moved this discussion to Lines 148-152.

• When discussing high-risk HPV (hrHPV), further clarity on which hrHPV strains are included is needed. This is important because:
o hrHPV strains are not universally distributed. While HPV 16/18 are prevalent globally and highly mutagenic, other hrHPV strains (e.g., hrHPV 52 and 58 in Central and South America) have higher prevalence and rates of cervical dysplasia/cancer in different regions.
o With increasing vaccination rates, clinicians are observing a decrease in HPV 16/18-related disease and an increasing prevalence of non-16/18 hrHPV-related precancerous and malignant lesions.
R: We thank the reviewer for this important observation. In the revised manuscript, we expanded the discussion on population-specific differences in cervicovaginal microbiome composition and highlighted that SMT may require adaptation to local microbial baselines, host factors, environmental exposures, and hrHPV genotype prevalence (Lines 104-111). We also clarified that SMT is proposed as a flexible conceptual framework that can be adapted to different hrHPV-associated settings.

Cervicovaginal Microbiome Dynamics
• The sentences in lines 79-82 are a bit awkward and require adjustment.
R: The text in lines 79-82 has been revised accordingly.

• Please be more specific about which Lactobacillus species corresponds to each Cervicovaginal State (CST) grouping. In fact the more recently updated CST or other systems are noted but not connected well to the review. Several arguments are made (e.g. lines 171, 178, 191, etc) but no solid conclusion drawn about what strains/species would be prioritized in SMT for phase 1 and 2. L. gasseri is highlighted (lines 171 -173) but potentially under-referenced. If multiple species/strains are to be included some mention of study of their synergism (or at least lack of competition) should be included.
R: We appreciate this insightful comment. In response, we clarified the relationships between CST classifications and dominant *Lactobacillus* species, expanded the rationale for selecting specific *Lactobacillus* species and strains for SMT, and incorporated additional discussion on the potential use of multi-strain formulations, including considerations regarding compatibility, synergistic effects, and microbial competition (Lines 232-239).

• For changes in the CVM, please clarify the timeframe over which these changes are observed (e.g., days, weeks, months, years). If changes occur over months to years, please additionally clarify how 'donor-dependent' (line 197) or the personalize approach would be applied to address the effects of hrHPV and expected changes from the menopausal transition, which can begin as early as the late 30s. Would CVM sampling over time be a critical component or is SMT based on a single snapshot before being initiated? This also seems the proper section to address directed changes in hygiene as part of SMT plans.
R: We appreciate the reviewer for raising this important point. We have expanded the discussion to clarify the temporal dynamics of the cervicovaginal microbiome and the potential need for longitudinal monitoring during SMT (Lines 334-336). We also discuss how individualized treatment strategies, including repeated microbiome assessments and consideration of factors such as menopause and vaginal hygiene practices, may influence the implementation and long-term success of SMT (Lines 341-344).

• The sentence contained in lines 227 to 229 should be adjusted and moved into this section, as it appears to relate to microbiome dynamics.
R: We relocated and revised the sentence from lines 227-229 so that it is discussed within the section on microbiome dynamics and temporal changes in the cervicovaginal ecosystem.

The Approach of Sequential Microbiome Therapy (SMT)
• Line 135 on indicates the bacteria that need to be inhibited leading to "disrupting these virus-bacterium feedback loops is therefore a critical goal of the first stage of SMT" but does not then declare the priority

Postal address
Department of Laboratory Medicine
Division of Biomolecular and Cellular Medicine
171 77 Stockholm, Sweden
Org. nummer 202100 2973

Visiting address
Alfred Nobels Allé 8
14152 Huddinge
Stockholm
Sweden

Contact
E-mail: **mariano.molina.beitia@ki.se**

approach. The authors posit “top strains” of Lactobacilli spp that lower vaginal pH but should then provide a table or review of the literature for those published strains and their properties if that is their conclusion for phase 2 approach. Others have reported different bacteria as disrupting the virus-bacteria connection but these are not noted. Figure 2 also seems to suggest multiple approaches for phase 1 consistent with a personalized medicine approach but the text fails to convey the specifics. Is the plan to offer oral/vaginal application of the 4 component blend shown in fig 2 for each condition? Are the Lactobacilli in phase 1 those used in phase 2? Again the lack of detail on the Lactobacilli spp reduces the advancement of the field ore refinements/suggestions for best study plans with this review.
R: We appreciate this detailed and constructive comment. In response, we expanded the discussion of Phase 1 and Phase 2 interventions and further justified the selection of *Lactobacillus* species and strains based on their reported biological properties (Lines 232-239).

• The sentence contained in lines 184-186 should be moved to an earlier point within this section.
R: We relocated and revised the sentence from lines 184-186 accordingly.

• Please clarify if your approach is able to differentiate between the regression of HPV infection and true clearance of the virus.
R: We appreciate the reviewer for raising this important point. We have clarified that SMT is a therapeutic framework and not intended to assess HPV outcomes directly (Lines 75-80, 176-180, 315-318, 348-353).

• Provide more detail on how this therapy is performed. Is it an oral or vaginal medication? Can it be administered at home or in a clinic setting, or will it require laboratory testing in advance?
R: We appreciate this important translational question. We have expanded the discussion to clarify that SMT is envisioned primarily as a vaginally administered intervention, potentially delivered through gels, capsules, sprays, or other locally applied formulations (Lines 201-203).

Advantages and Limitations
• For the preclinical data discussed in lines 206-209, please clarify if regions other than Africa and North America were included in the analysis. Additionally, please specify which country in North America the samples were obtained from. Ultimately a commentary on SMT application in distinct populations would be of value.
R: We appreciate the reviewer for raising this important consideration. We have clarified the description of the cited study (Line 304) and expanded the discussion on population-specific differences in cervicovaginal microbiome composition, emphasizing that SMT may ultimately require adaptation to distinct geographic, ethnic, and epidemiological contexts (Lines 104-111).

• Beyond the need for further testing in other clinical settings, please include additional limitations. For example, clarify if SMT would be useful for "late" HPV disease.
Additional references that should be included or at least noted:
1) https://journals.asm.org/doi/10.1128/msystems.00017-22
2) https://journals.asm.org/doi/10.1128/mbio.02677-25?utm_source=TOC&utm_medium=ealert&utm_campaign=TOC_v17_i3&ref=16e4f3c1-40d6-4d96-804e-6cf3d92cfda5
R: We appreciate this valuable suggestion. In response, we expanded the discussion to further acknowledge the limitations of SMT, including the uncertainty regarding the intended use and its likely greatest utility during earlier stages of disease progression (Box 1).

Figures and Acronyms
• For Figure 1, it would be helpful to include the specific names of the Lactobacillus strains mentioned.
• For Figure 2, "BDA" is not defined in either the figure or the text (biofilm-disrupting agent?).
R: We thank the reviewer for these helpful suggestions. We have revised Figure 1 to more clearly indicate the *Lactobacillus* species relevant to SMT and updated the Figure 2 legend to define BDA as biofilm-disrupting

agents, along with the other abbreviations used throughout the figure.

Reviewer #5 (Remarks to the Author):

I co-reviewed this manuscript with one of the reviewers who provided the listed reports. This is part of the Communications Biology initiative to facilitate training in peer review and to provide appropriate recognition for Early Career Researchers who co-review manuscripts.

Postal address
Department of Laboratory Medicine
Division of Biomolecular and Cellular Medicine
171 77 Stockholm, Sweden
Org. nummer 202100 2973

Visiting address
Alfred Nobels Allé 8
14152 Huddinge
Stockholm
Sweden

Contact
E-mail: **mariano.molina.beitia@ki.se**

Reviewer #1 (Remarks to the Author):
The authors have addressed and complied with every single point raised by myself and the four additional Reviewers that assessed their work in a satisfactory fashion. Therefore, I recommend the acceptance of this revised manuscript in its current form.
R: We thank the reviewer for the positive assessment of our revised manuscript and for recommending acceptance.

Reviewer #2 (Remarks to the Author):
The authors have addressed the points raised and I have no further requests, except to replace the word "may" with "drastically" in the following sentence referring to pharma-grade manufacturing cost in lines 318-318 : " These requirements may increase development costs and.."
R: We thank the reviewer for this suggestion. We have revised the sentence accordingly to state that these requirements “drastically increase development costs.” (Line 331)

Reviewer #5 (Remarks to the Author):
I co-reviewed this manuscript with one of the reviewers who provided the listed reports. This is part of the Communications Biology initiative to facilitate training in peer review and to provide appropriate recognition for Early Career Researchers who co-review manuscripts.
R: We thank the reviewer for their contribution to the review process.

Reviewer #6 (Remarks to the Author):
As a newly-added reviewer after the first round of review, I think the authors in general address the comments very well. I only have a few comments on the authors' response to the second comment of reviewer #1, which is about the "Phase 1" of the SMT on colonization resistance.

In the response the authors expanded the discussion of potential barriers to successful SMT phase 1 implementation, including biofilm colonization resistance and possible supplements of glycogen or milk oligosaccharides. One thing that I am not sure about the phase 1, however, is the agent that is used to inhibit the potential detrimental bacteria. In Figure 1 I can see that there are compounds such as BDA, Cysteine inhibitor, antibiosis, and live bacteria such as Lactobacillus strains. Putting compounds and bacterial strains together, however, may be a bit misleading. While the compounds are known to inhibit bacteria, the effect of the introduction of Lactobacillus strains is not so certain. Yes I know that the authors discuss some of the potential effects of the Lactobacillus in Line 221-229; but the introduction of Lactobacillus strains in Phase 1 almost feels like Phase 2 is not necessary at all. Please carefully think about whether it is really necessary to put Lactobacillus strains in Phase 1 for disruption purpose.

There is one more thing that may need the authors' attention: the potential risk of probiotics. As was discussed in Pandey 2025 (doi:10.1017/S0022029925101155), there are potential risks of administered antibiotics such as horizontal gene transfer, antimicrobial resistance genes, or even infectious diseases caused by the probiotics. Although the introduction of Lactobacillus species into the vaginal environment is not conventional "probiotics", broadly speaking it still roughly falls into this definition. Some discussion would be very helpful in the control of potential risks.
R: We thank the reviewer for these thoughtful comments. We agree that the previous presentation could have made the respective roles of *Lactobacillus* strains in Phases 1 and 2 insufficiently clear. We have therefore revised Figure 1 and its legend and added clarification in the main text that the distinction between the two phases is functional. Strains used in Phase 1 would be selected primarily for their ability to disrupt dysbiosis-associated communities, whereas Phase 2 would prioritize durable engraftment and long-term ecological stability; the different or same strain could potentially contribute to two different phases. We have also clarified in Figure 2 that bacterial strains used in Phase 1 would be selected for inhibitory activity (Lines 189-197, 237-241, 245).

Postal address
Department of Laboratory Medicine
Division of Biomolecular and Cellular Medicine
171 77 Stockholm, Sweden
Org. nummer 202100 2973

Visiting address
Alfred Nobels Allé 8
14152 Huddinge
Stockholm
Sweden

Contact
E-mail: **mariano.molina.beitia@ki.se**

We also agree that the safety of live bacterial administration should be discussed more explicitly. We have expanded the manuscript to note the need for strain-level safety assessment, including evaluation of transferable antimicrobial resistance determinants, virulence-associated traits, and infectious risks (Lines 280-281). We further added discussion of rare invasive infections associated with probiotic *Lactobacillus* administration, particularly in vulnerable populations, and cited Pandey et al. (2025) accordingly (Lines 332-335).

Postal address
Department of Laboratory Medicine
Division of Biomolecular and Cellular Medicine
171 77 Stockholm, Sweden
Org. nummer 202100 2973

Visiting address
Alfred Nobels Allé 8
14152 Huddinge
Stockholm
Sweden

Contact
E-mail: **mariano.molina.beitia@ki.se**
